# LncRNA LOC105378097 inhibits cardiac mitophagy in natural ageing mice

**DOI:** 10.1002/ctm2.908

**Published:** 2022-06-27

**Authors:** Xin Liu, Xue Bai, Heng Liu, Yang Hong, Hao Cui, Lei Wang, Wanqing Xu, Limin Zhao, Xiaohan Li, Huimin Li, Xia Li, Hui Chen, Ziyu Meng, Han Lou, Henghui Xu, Yuan Lin, Zhimin Du, Philipp Kopylov, Baofeng Yang, Yong Zhang

**Affiliations:** ^1^ Department of Pharmacology (The State‐Province Key Laboratories of Biomedicine‐Pharmaceutics of China, Key Laboratory of Cardiovascular Research, Ministry of Education), College of Pharmacy Harbin Medical University Harbin China; ^2^ Department of Pharmacology and Therapeutics, Melbourne School of Biomedical Sciences, Faculty of Medicine Dentistry and Health Sciences University of Melbourne Melbourne Australia; ^3^ Research Unit of Noninfectious Chronic Diseases in Frigid Zone Chinese Academy of Medical Sciences Harbin China; ^4^ Institute of Metabolic Disease Heilongjiang Academy of Medical Science Harbin China; ^5^ Institute of Clinical Pharmacy The Second Affiliated Hospital of Harbin Medical University Harbin China; ^6^ Department of Preventive and Emergency Cardiology Sechenov First Moscow State Medical University Moscow Russian Federation

**Keywords:** cardiac function, heart ageing, LOC105378097, mitophagy, Parkin

## Abstract

**Background:**

The development of heart ageing is the main cause of chronic disability, disease and death in the elderly. Ample evidence has established a pivotal role for significantly reduced mitophagy in the ageing heart. However, the underlying mechanisms of mitophagy deficiency in ageing heart are little known. The present study aimed to explore the underlying mechanisms of lncRNA LOC105378097 (Senescence‐Mitophagy Associated LncRNA, lncR‐SMAL) actions on mitophagy in the setting of heart ageing.

**Methods:**

The expression of lncR‐SMAL was measured in serum from different ages of human and heart from different ages of mice through a quantitative real‐time polymerase chain reaction. The effects of lncR‐SMAL on heart function of mice were assessed by echocardiography and pressure‐volume measurements system. Cardiac senescence was evaluated by hematoxylin‐eosin staining, senescence‐associated β‐galactosidase staining, flow cytometry and western blot analysis of expression of ageing related markes p53 and p21. Cardiomyocyte mitophagy was assessed by western blot, mRFP‐GFP‐LC3 adenovirus particles transfection and mito‐Keima staining. Interaction between lncR‐SMAL and Parkin was validated through molecular docking, RNA immunoprecipitation (RIP) and RNA pull‐down assay. Ubiquitination assay was performed to explore the molecular mechanism of Parkin inhibition. The effects of lncR‐SMAL on mitochondrial function were investigated through electron microscopic examination, JC‐1 staining and oxygen consumption rates analysis.

**Results:**

The heart‐enriched lncR‐SMAL reached the expression crest in the serum of human at an age of 60. Exogenously overexpression of lncRNA SMAL deteriorated cardiac function exactly as natural ageing and inhibited the associated cardiomyocytes mitophagy by depressing Parkin protein level. Improved heart ageing and mitophagy caused by Parkin overexpression were reversed by lncR‐SMAL in mice. In contrast, the loss of lncR‐SMAL in AC16 cells induced the upregulation of Parkin protein and ameliorated mitophagy and mitochondrial dysfunction, resulting in alleviated cardiac senescence. Besides, we found the interaction between lncR‐SMAL and Parkin protein through computational docking analysis, pull‐down and RIP assay. This would contribute to the promotive effect of lncR‐SMAL on Parkin ubiquitination and decrease Parkin protein stability.

**Conclusions:**

The present study for the first time demonstrates a heart‐enriched lncRNA, SMAL, that inhibits the mitophagy of cardiomyocytes via the downregulation of Parkin protein, which further contributes to heart ageing and cardiac dysfunction in natural ageing mice.

## Graphical abstract

(1) The heart‐enriched lncR‐SMAL is robustly increased in its level in serum samples of human subjects over 60‐year old relative to young controls, and in both nucleus and cytoplasm of senescent cardiomyocytes. (2) Artificial overexpression of lncR‐SMAL induces cardiac senescence by inhibiting Parkin‐mediated mitophagy. Knockdown of endogenous lncR‐SMAL in cardiomyocytes partially abrogated cell senescence. (3) LncR‐SMAL interacts with Parkin protein and promotes ubiquitin‐proteasome degradation of Parkin in senescent cardiomyocytes.

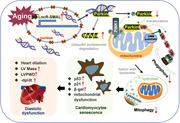

## 1 INTRODUCTION

Cardiovascular disease (CVD) seriously threatens the health and quality of life of the elderly, and the incidence increases with age. Increasing age causes a progressive deterioration of tissues and organs, leading to an enhancement of vulnerability to stress. Although efforts to improve the outcome of circulatory diseases have led to the decrease of the mortality, they are still the leading cause of death worldwide.^1^ Ageing is the main risk factor and a burden for healing in CVDs,^2^ which will have a substantial impact on the costs of health care system.^3^

Transcriptomic data reveal that <3% of the transcriptome is protein‐coding genes.^4^ The rest are non‐coding RNAs (ncRNAs) with limited coding capacity, including microRNAs, transfer RNAs, small nucleolar RNAs and long ncRNAs (lncRNAs or lncRs).^5^, ^6^ LncRNAs are defined as transcripts of more than 200 nt in length.^7^ The action mechanisms of lncRNAs are extremely diverse, including transcriptional regulation, epigenetic regulation, and post‐transcriptional regulation.^8^ Most of these functions are exerted by binding of the lncRNA to proteins, such as transcription factors, chromatin modifiers and other functional proteins. In the last decade, an increasing number of studies indicate the crucial roles of lncRNAs in development and diseases, including ageing and CVDs.^9^, ^10^, ^11^, ^12^, ^13^, ^14^ Our previous studies also revealed that lncRNA could directly bind to target protein, exerting effects on calcium homeostasis and electrical conduction in myocardial infarction and arrhythmia, respectively.^13^, ^15^, ^16^, ^17^ In cardiovascular senescence, lncRNAs are arising as a new frontier for treatment therapy.^18^, ^19^, ^20^ The biggest limitation about research and application of lncRNAs is the lack of tissue specificity. Hence we first screened the heart‐enriched lncRNAs in GenBank and detected their expressions in ageing. Among them, LOC105378097 showed the highest expression in senescent AC16 cells. The pathophysiological functions and molecular mechanisms of LOC105378097 were totally unclear. Therefore, we further investigated the role of LOC105378097 in heart ageing.

Mitochondria are important for cell metabolism and cell survival.^21^, ^22^ Mitochondrial dysfunction and reduced mitochondrial content are associated with ageing and related pathophysiological conditions.^23^ The damaged mitochondria not only produce excessive amounts of reactive oxygen species but also stimulate cell death pathway.^24^ Removal of the damaged mitochondria is critical to maintain cell survival and protect cells from injury. Mitophagy is the process that selectively removes and degrades dysfunctional mitochondria via autophagy and has been shown to be protective in several CVDs, including ischaemia‐reperfusion,^25^ heart failure^26^, ^27^ and heart ageing.^28^ Several studies have demonstrated that the enhancement of mitophagy prevents cardiac injury caused by stress and other damage sources, whereas the impairment of mitophagy results in the accumulation of dysfunctional mitochondria and increased tissue damage.^26^ The senescence‐associated impairment of mitophagy has been reported.^29^ Inhibition of mitophagy in the heart induces age‐related cardiomyopathy.^30^ Mitophagy‐related genes are associated with lifespan in worms and flies.^31^, ^32^ Although the functional role of mitophagy in ageing has been revealed, the upstream triggers for declining mitophagy as age increases still need further investigation.

Here we utilized both in vivo and in vitro models of heart ageing to test how age influenced mitophagic response and whether heart‐specific lncRNA LOC105378097 was the key controller. For the sake of convenience, we named it SMAL (Senescence‐Mitophagy Associated LncRNA) or lncR‐SMAL.

## 2 RESULTS

### 2.1 LncR‐SMAL is dysregulated in cardiac senescence

As the first step towards understanding the potential roles of lncRNAs in cardiac senescence, we screened a subset of lncRNAs for their differential expressions between d‐gal‐treated and non‐treated control AC16 cells using quantitative real‐time PCR (qRT‐PCR). Based on the NCBI database, 22 human lncRNAs were annotated as cardiac‐specific or enriched lncRNAs and therefore were included in our initial screening list. These lncRNAs included LOC101927404, LOC101927145, LOC107986043, LOC102724319, LOC102723850, LOC101928359, LINC02517, LOC107986462, LOC107986203, LOC105379404, LOC105372686, LINC02208, LINC01479, LOC105379003, LOC105378351, LOC105373170, LOC105370446, LOC101929258, LOC105371646, LOC105369998, LOC101927072 and LOC105378097 (lncRNA SMAL or lncR‐SMAL). The results showed that, compared with the relative control, LOC105378097 was the most elevated in d‐gal‐induced senescent AC16 cells (Figure S1A). To further test the enrichment of lncR‐SMAL in different organs, we detected the expression level of lncR‐SMAL in heart, liver, spleen, lung, kidney, fat, muscle, aorta and pancreas of wild‐type C57BL/6 mice. The results showed that lncR‐SMAL was enriched in heart compared to other tested organs (Figure S1B). Moreover, the expression of lncR‐SMAL in different cell types of heart was also detected. As shown in Figure S1C, lncR‐SMAL was highly expressed in cardiomyocytes. Therefore, we concluded that lncR‐SMAL was heart enrichment or cardiomyocytes enrichment.

**FIGURE 1:**
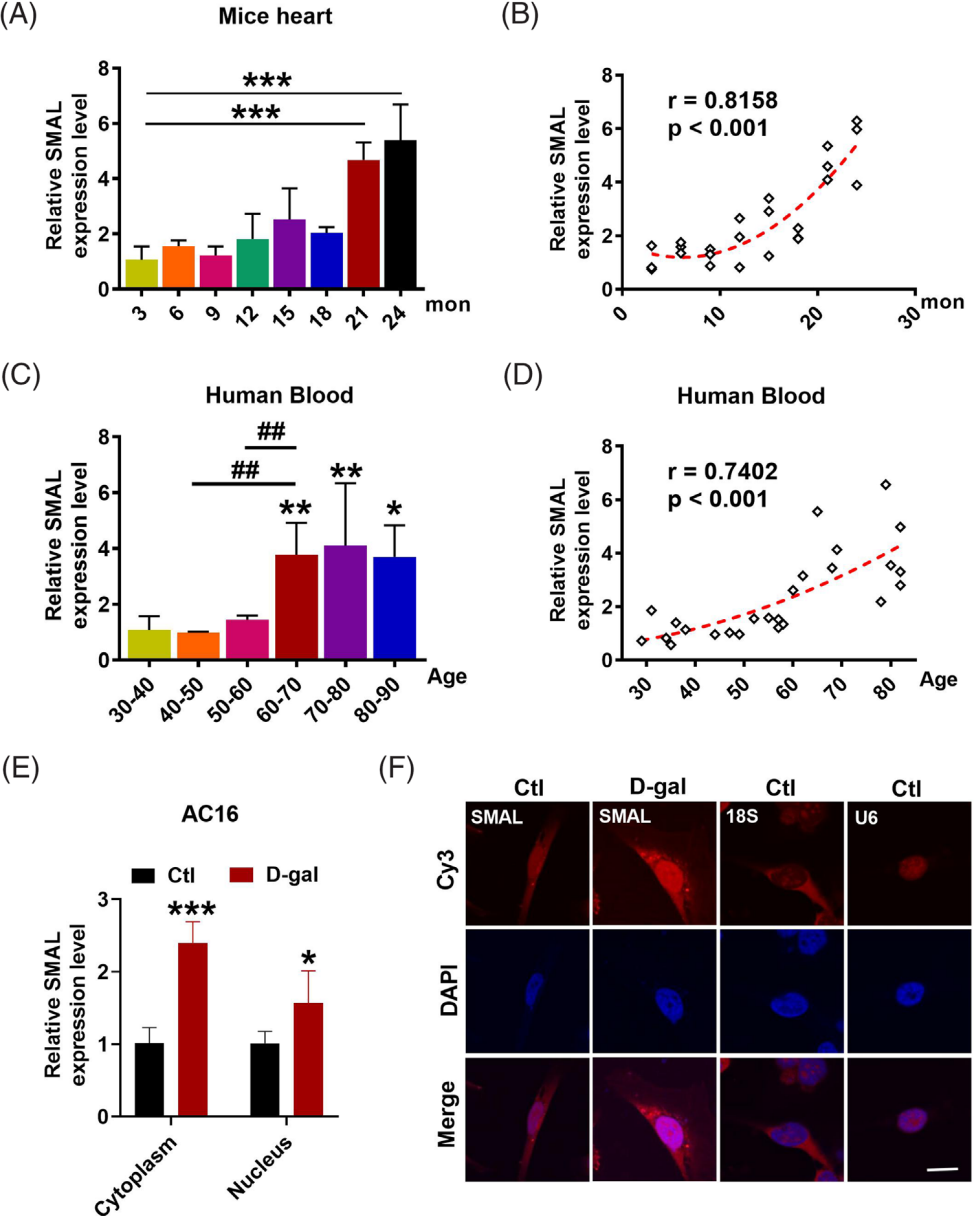
LncR‐SMAL is dysregulated in heart ageing. (A and B) Expression levels of lncR‐SMAL in hearts from different ages of mice tested by quantitative real‐time PCR (qRT‐PCR); *n* = 3. ***p < .001 versus 3 month. (C and D) Expression levels of lncR‐SMAL in different ages of human blood; age 30–40, *n* = 6; age 40–50, *n* = 3; age 50–60, *n* = 5; age 60–70, *n* = 5; age 70–80, *n* = 3; age 80–90, *n* = 3. *p < .05, **p < .01 versus age 30–40; ^##^ *p* < .01, compared with groups at both ends of the line. (E) Expression levels of lncR‐SMAL in nucleus and cytoplasm of AC16 cells tested by qRT‐PCR; *n* = 5. *p < .05, ***p < .001 versus Ctl. (F) Subcellular distribution of lncR‐SMAL in AC16 cells with or without d‐gal determined by fluorescence in situ hybridization (FISH). 18S and U6 were used as markers for cytoplasm and nucleus, respectively. Scale bar: 20 μm; *n* = 5. The data were expressed as the mean ± SD.

We then investigated the connection between lncR‐SMAL and ageing. The differential expression of lncR‐SMAL was detected in hearts from different ages of mice. The results showed that lncR‐SMAL was remarkably elevated in mice over 21‐month old, and lncR‐SMAL level was highly and positively correlated with the age of mice with a correlation coefficient of .8158 (Figure 1A,B). To validate the observations obtained from mice heart, we quantified the possible age differences in lncR‐SMAL levels in human blood. As anticipated, lncR‐SMAL levels were remarkably higher in serum samples of aged patients (>60‐year old) than of young control subjects (*r* = .7412) (Figure 1C,D). We then found lncR‐SMAL was expressed both in cytoplasm and nucleus of cardiomyocytes, whereas d‐gal treatment (40 μM) increased lncR‐SMAL level both in cytoplasm and nucleus (Figure 1E,F). These results suggested lncR‐SMAL as an ageing heart‐upregulated lncRNA.

### 2.2 LncR‐SMAL impairs cardiac diastolic function and mitophagy in mice

**FIGURE 2:**
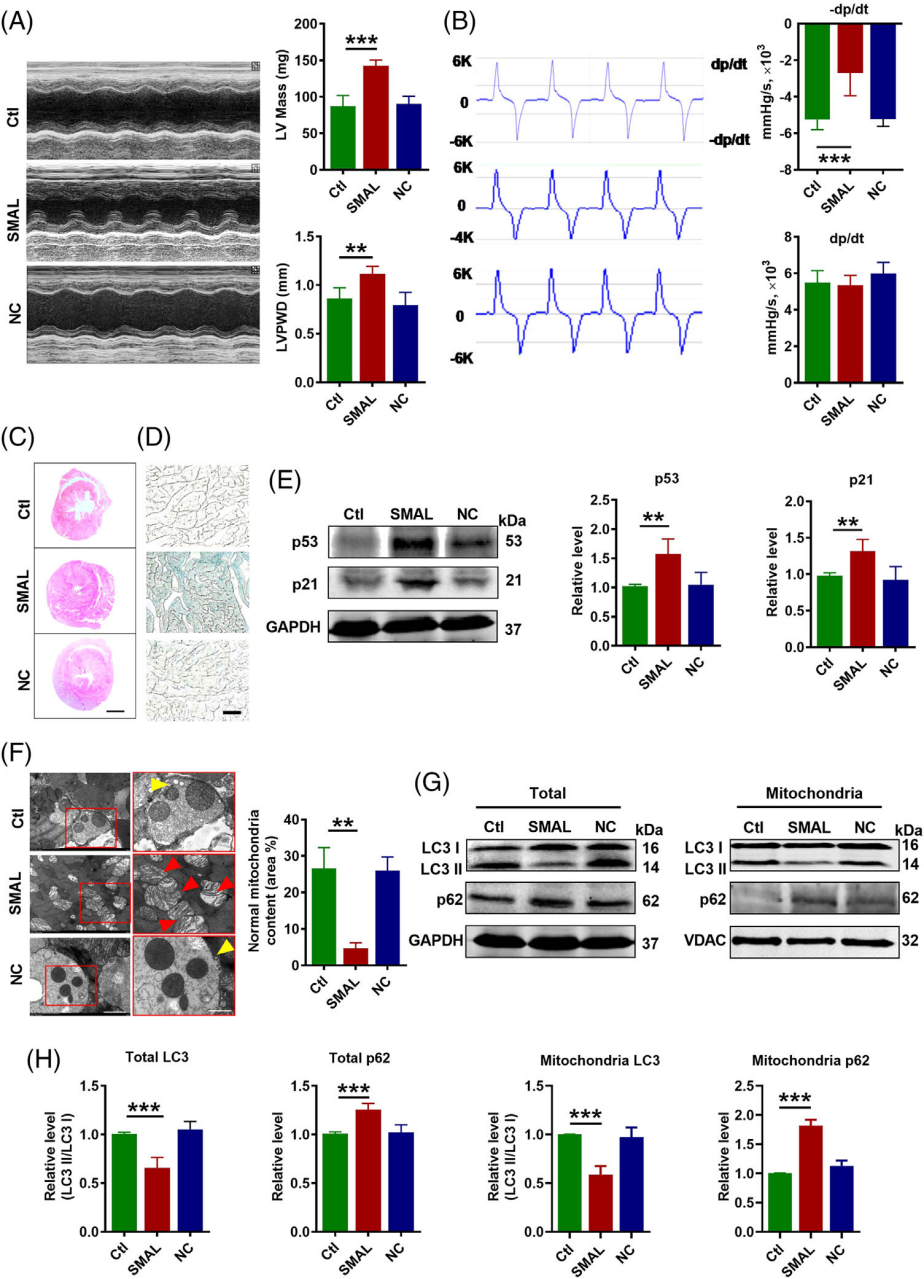
LncR‐SMAL impairs cardiac diastolic function and cardiac remodelling: (A) Left ventricular mass (LV mass) and diastolic left ventricular posterior wall thickness (LVPWD) measured by echocardiography. *n* = 5. **p < .01, ***p < .001 versus Ctl. (B) ±*dp*/*dt*_max_ measured by pressure–volume measurements system; *n* = 5. ***p < .001 versus Ctl. (C) Histological sections stained with HE. Scale bar: 500 μm; *n* = 3. (D) Cardiac senescence tested by β‐galactosidase staining. Scale bar: 5 μm; *n* = 5. (E) p53 and p21 protein levels tested by western blot; *n* = 5. **p < .01 versus Ctl. (F) Mitophagy detected by electron microscopy in cardiac tissue. The yellow arrow represents mitophagy vacuole; red arrow represents damaged mitochondria. Scale bar:   2 and 1 μm ; *n* = 3. (G and H) Light chain 3 (LC3) and p62 protein levels in total protein and mitochondria tested by western blot; *n* = 5. ***p < .001 versus Ctl. The data were expressed as the mean ± SD.

A question we asked was whether upregulation of lncR‐SMAL in mice was a contributor to heart ageing or was merely a bystander. To clarify this issue, we investigated the effects of lncR‐SMAL overexpression on cardiac function and heart ageing of wide‐type mice through the infection of adeno‐associated virus (AAV) 9‐SMAL. After 3 months of treatment, the overexpression of lncR‐SMAL in different organs of mice was confirmed (Figure S1B), and the results showed a large increase of lncR‐SMAL in heart of mice. We then measured cardiac function using echocardiography and pressure–volume measurements system. The results showed that lncR‐SMAL overexpression weakened cardiac diastolic function, as indicated by increased left ventricular mass (LV Mass) and diastolic LV posterior wall thickness (LVPWD) (Figure 2A). The maximum rate of drop of LV pressure during relaxation (−*dp*/*dt*_max_) has been considered the better manifestation of cardiac relaxation functions and was decreased with lncR‐SMAL overexpression. In contrast, systolic function parameters including the maximum rate of rise of LV pressure during systolic (+*dp*/*dt*_max_), and ejection fraction (EF) and fractional shortening (FS) were unaffected by lncR‐SMAL overexpression (Figures 2B andS2A). Depressed cardiac diastolic function by lncR‐SMAL suggested that this lncRNA might cause pathological cardiac structural remodelling. To test this notion, we performed HE staining in the cross section of the whole heart. As illustrated in Figure 2C, the heart was significantly enlarged in mice with lncR‐SMAL overexpression compared to those without lncR‐SMAL treatment. Intriguingly, the impaired diastolic function and hypertrophied heart caused by lncR‐SMAL were similar to the degenerative phenotypes of the heart in natural ageing mice.^33^, ^34^ Moreover, heart ageing is associated with the upregulation of senescence‐associated secretory phenotype (SASP). Compared with control hearts, lncR‐SMAL overexpression hearts showed significant increases in SASP, including and TNF‐α, IL‐6, IL‐1, MMP‐2 and MMP‐9 (Figure S2B). We went on to investigate the effects of lncR‐SMAL on cardiac senescence. A marked increase in the positive staining of the heart with senescence‐associated β‐galactosidase (SA‐β‐Gal) was shown in the lncR‐SMAL group (Figure 2D). Then we observed that the protein levels of senescence marker genes p53 and p21 were both substantially increased by lncR‐SMAL (Figure 2E). To elucidate the subcellular mechanisms of lncR‐SMAL's action, we used transmission electronic microscopy (TEM) to examine the ultrastructural changes in the cardiac tissue (Figure 2F). The images revealed that lncR‐SMAL overexpression resulted in swelling, damaged cardiomyocyte mitochondria and mitigation of mitophagy. In response to mitophagy, microtubule‐associated protein 1 light chain 3‐I (LC3 I) is conjugated to phosphatidylethanolamine to form LC3‐phosphatidylethanolamine conjugate (LC3 II), which is recruited to autophagosomal membranes. The protein sequestosome 1 (SQSTM1/p62) is able to bind ubiquitin and also LC3, thereby targeting the autophagosome and facilitating clearance of ubiquitinated proteins. We then evaluated mitophagy level by the detection of LC3 II/I and p62 expression levels in total cell lysate and mitochondrial content. Western blot results further confirmed the decreased autophagy or mitophagy with the downregulation of LC3 II/I and elevation of autophagosome adaptor protein p62 both in total cell lysate and mitochondria (Figure 2G,H). These results suggested that lncR‐SMAL inhibited mitophagy in heart ageing.

### 2.3 LncR‐SMAL downregulates Parkin expression in hearts of ageing mice

**FIGURE 3:**
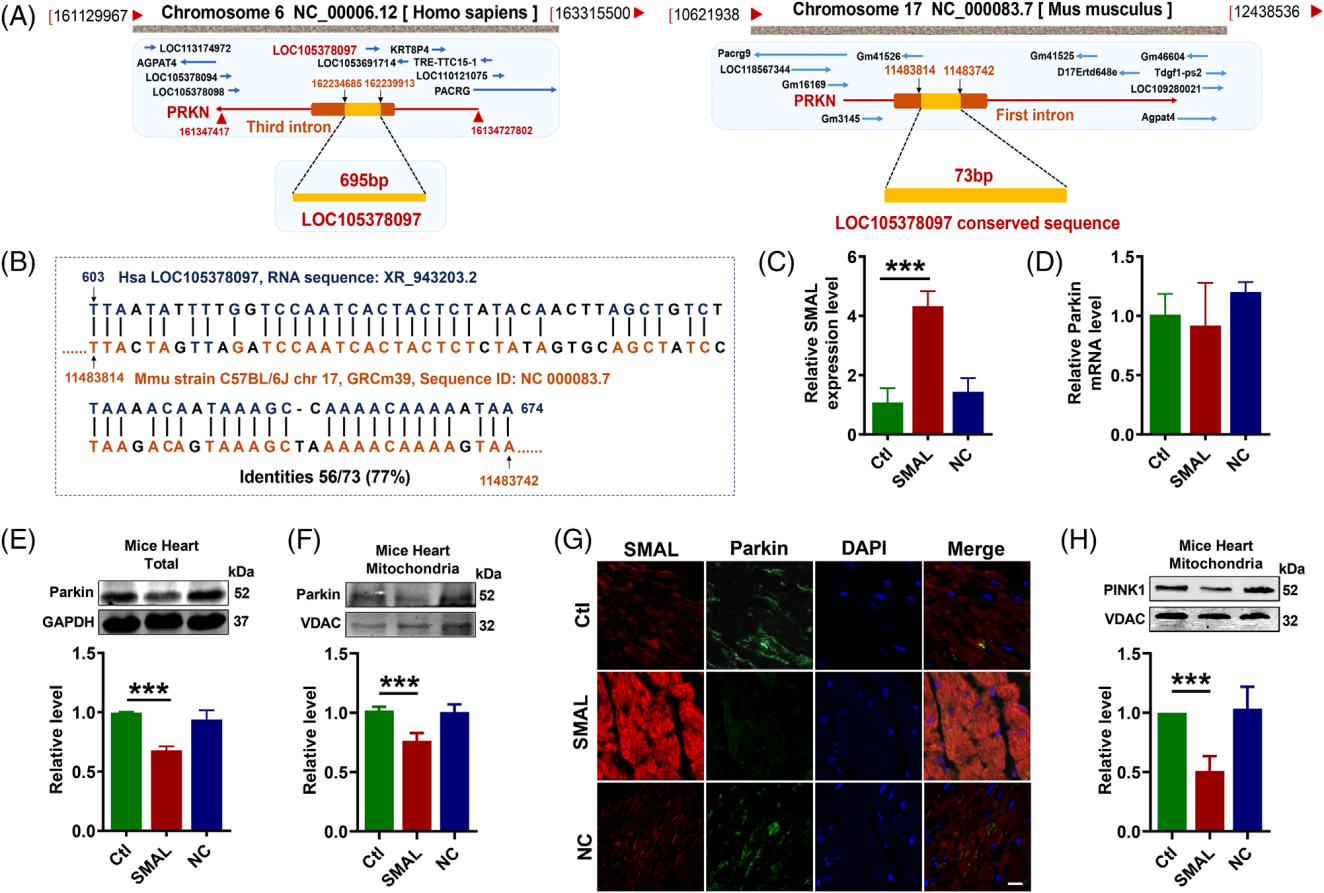
LncR‐SMAL downregulates Parkin in mouse and human hearts. (A) Schematic diagram for genome information of lncR‐SMAL and lncR‐SMAL conserved sequence in home sapiens and Mus musculus, respectively. (B) Sequence of lncR‐SMAL conserved sequence in human and mouse genome identified by NCBI BLAST database. (C and D) LncR‐SMAL and Parkin mRNA levels tested by quantitative real‐time PCR (qRT‐PCR), *n* = 5. ***p < .001 versus Ctl. (E and F) Parkin protein levels in total protein and mitochondria in hearts of mice tested by western blot; *n* = 5. ***p < .001 versus Ctl. (G) Expression of lncR‐SMAL (red) and Parkin (green) protein detected by fluorescence in situ hybridization (FISH) and immunofluorescent staining. The nuclei (blue) stained with DAPI. Scale bar: 20 μm; *n* = 3. (H) PINK1 protein level in mitochondria in hearts of mice tested by western blot; *n* = 5. ***p < .001 versus Ctl. The data were expressed as the mean ± SD.

To investigate the downstream target of lncR‐SMAL, we subsequently analysed the genome structure and sequence of lncR‐SMAL using GenBank, UCSC and Ensemble databases. Human lncR‐SMAL is located on chromosome 6, and it is transcribed from the third intron of PRKN (Parkin) gene (Figure 3A). However, the mouse lncR‐SMAL (mmu‐lncR‐SMAL) sequence has not been identified in GenBank. We then blasted the human lncR‐SMAL sequence with mouse genomic plus transcript (mouse G + T) using NCBI BLAST database. As shown in Figure 3A,B, conserved sequence with a length of 73 nt was found on the first intron of chromosome 17 in mouse genome. To identify the transcription of lncR‐SMAL in mouse, we synthesized three pairs of primers based on this conversed sequence in mouse genome. Total RNA was extracted from neonatal mouse primary cardiomyocytes. Amplified product after reverse transcription was detected by southern blot (Figure S3A), and the most enriched products were sequenced by ABI3730 Sequencing Instrument with dideoxy end termination method. We then blasted the sequencing nucleotide with mouse genomic plus transcript (mouse G + T) (Figure S3B). As expected, the amplified cDNA product strictly fits with mouse genome with identities of 100%. Based on these results, we proved the transcription of lncR‐SMAL from mouse genome. Notably, the successful detection of lncR‐SMAL expression by qRT‐PCR in mice heart has also been confirmed. However, the whole sequence of mmu‐lncR‐SMAL still needs further investigation. Several studies have reported the unique regulatory connection between lncRNA and its neighbouring or host gene. Therefore, we tested the possible regulatory mechanism of lncR‐SMAL and Parkin. Both western blot and immunofluorescence staining results demonstrated that the exogenous overexpression of lncR‐SMAL remarkably suppressed Parkin protein levels no matter in total cell lysate and mitochondrial content of in mouse heart (Figure 3C,E–G). However, the mRNA level of Parkin was not influenced by an overexpression of lncR‐SMAL (Figure 3D). Phosphatase and tensin homolog (PTEN)‐induced putative kinase 1 (PINK1) is the upstream activating kinase of Parkin and is stabilized and accumulates in depolarized mitochondria to initiate mitophagy.^35^ We found lncR‐SMAL also induced the reduction in PINK1 protein in heart mitochondria (Figure 3H). To further confirm the regulatory roles of lncR‐SMAL in cardiomyocytes, we detected cell mitophagy and senescence in neonatal mouse primary cardiomyocytes after the overexpression of lncR‐SMAL. The results were consistent with the in vivo data and confirmed that lncR‐SMAL overexpression remarkably impaired mitophagy and promoted senescence (Figure S4A,B).

### 2.4 LncR‐SMAL directly binds to Parkin protein

**FIGURE 4:**
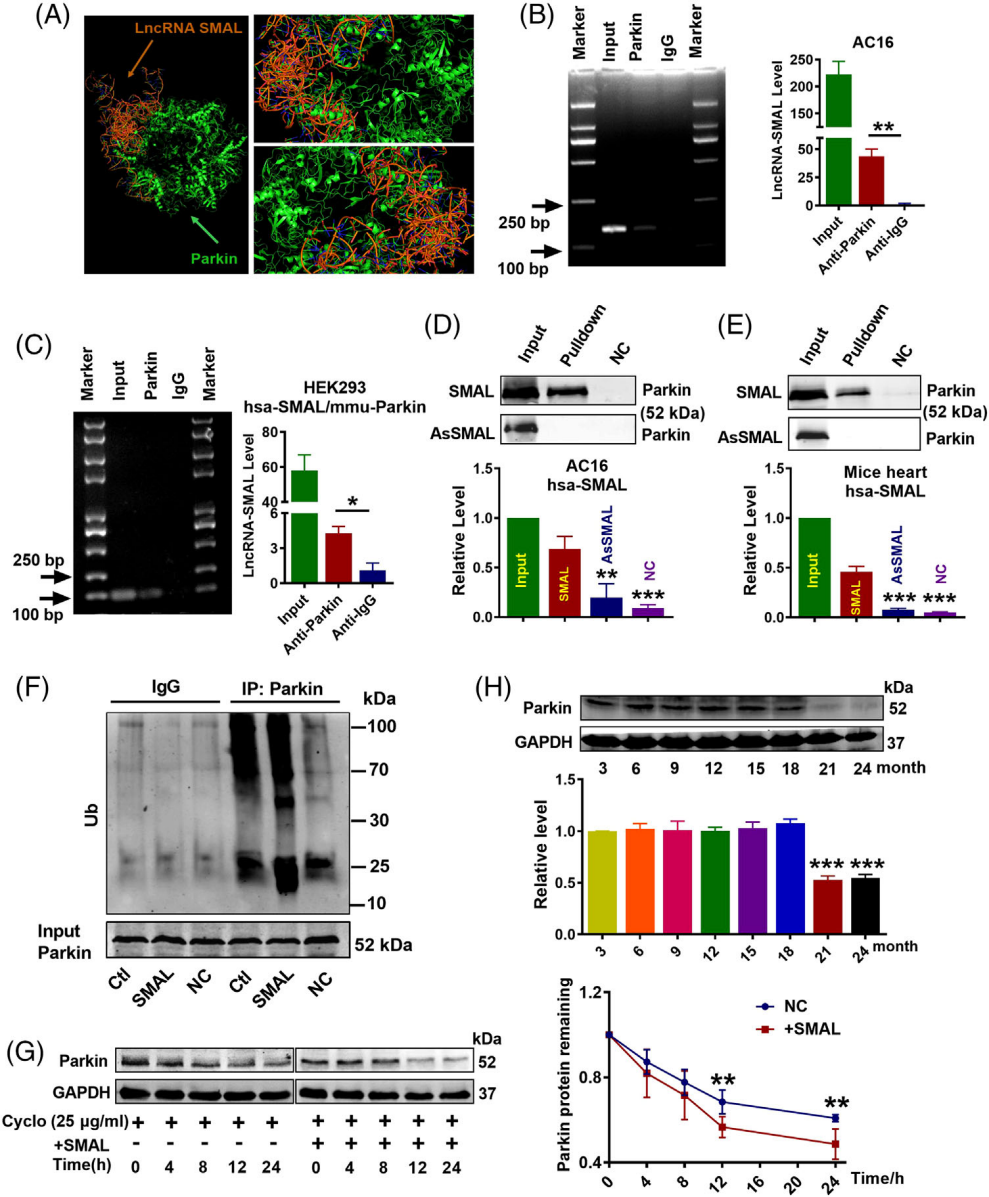
Interaction between lncR‐SMAL and Parkin protein. (A) Left panel: theoretical prediction and analysis of direct lncR‐SMAL:Parkin binding using the HEX8.0/Pymol docking software; right panels: enlarged renderings. (B) RNA‐binding protein immunoprecipitation (RIP) analysis for lncR‐SMAL:Parkin interaction in AC16 cells; *n* = 3. (C) RIP analysis for lncR‐SMAL:Parkin interaction in HEK293 cells with cotransfection of hsa‐lncR‐SMAL and mmu‐Parkin plasmid; *n* = 3. (D and E) RNA pulldown of lncR‐SMAL dragged down an appreciable quantity of Parkin in AC16 cells and heart tissue of mouse, respectively. An antisense fragment to lncR‐SMAL (AsSMAL) was used; *n* = 3. **p < .01, ***p < .001 versus SMAL. (F) Ubiquitin of Parkin detected by co‐immunoprecipitation (CO‐IP) assay, *n* = 3. (G) Parkin protein level tested by western blot, the protein synthesis inhibitor cycloheximide (Cyclo) (25 μg/ml) used; *n* = 5. **p < .01 versus negative control (NC). (H) Parkin protein level in heart tissue from different ages of mice tested by western blot; *n* = 3. ***p < .001 versus 3 month. the data were expressed as the mean ± SD.

We then used the RNA‐Protein Interaction Prediction (RPISeq) database to analyse the binding potent between hsa‐lncR‐SMAL and hsa‐Parkin protein. The predicted score indicated a high affinity for their interaction (RF classifier: .8; SVM classifier: .74). Moreover, HEX8.0/Pymol software simulated the possible binding configuration between the two molecules (Figure 4A). Moreover, the interaction between lncR‐SMAL and PINK1 was also analysed and with negative results (data not shown). We therefore used RNA‐binding protein immunoprecipitation (RIP) to confirm whether lncR‐SMAL could physically bind to human and mouse Parkin protein. The results depicted in Figure 4B,C clearly indicated the immuno‐precipitation of Parkin carried an appreciable amount of lncR‐SMAL, no matter human and mouse Parkin proteins. Additionally, the binding affinity was also confirmed in senescent AC16 cells (Figure S5A). Similarity, RNA pull‐down of lncR‐SMAL dragged down an appreciable quantity of Parkin both in AC16 cells and mouse heart tissues (Figure 4D,E). These results suggested that lncR‐SMAL had a strong affinity to Parkin both in human and mouse. Demonstration of the interaction between lncR‐SMAL and Parkin led us to examine whether or not lncR‐SMAL promotes the ubiquitination of Parkin. The results showed that Parkin ubiquitination level was markedly increased in lncR‐SMAL overexpressed AC16 cells (Figure 4F). Subsequently, increased ubiquitination of Parkin significantly decreased Parkin protein stability (Figure 4G). The role of Parkin in ageing has been reported, but the age‐related characteristics of Parkin expression have not been clearly clarified.^28^, ^36^ Our results exhibited that the protein level of Parkin in cardiomyocytes was age related and decreased abruptly in mice of 21‐month (Figure 4H).

### 2.5 LncR‐SMAL impairs Parkin‐mediated improvement of cardiac function in aged mice

**FIGURE 5:**
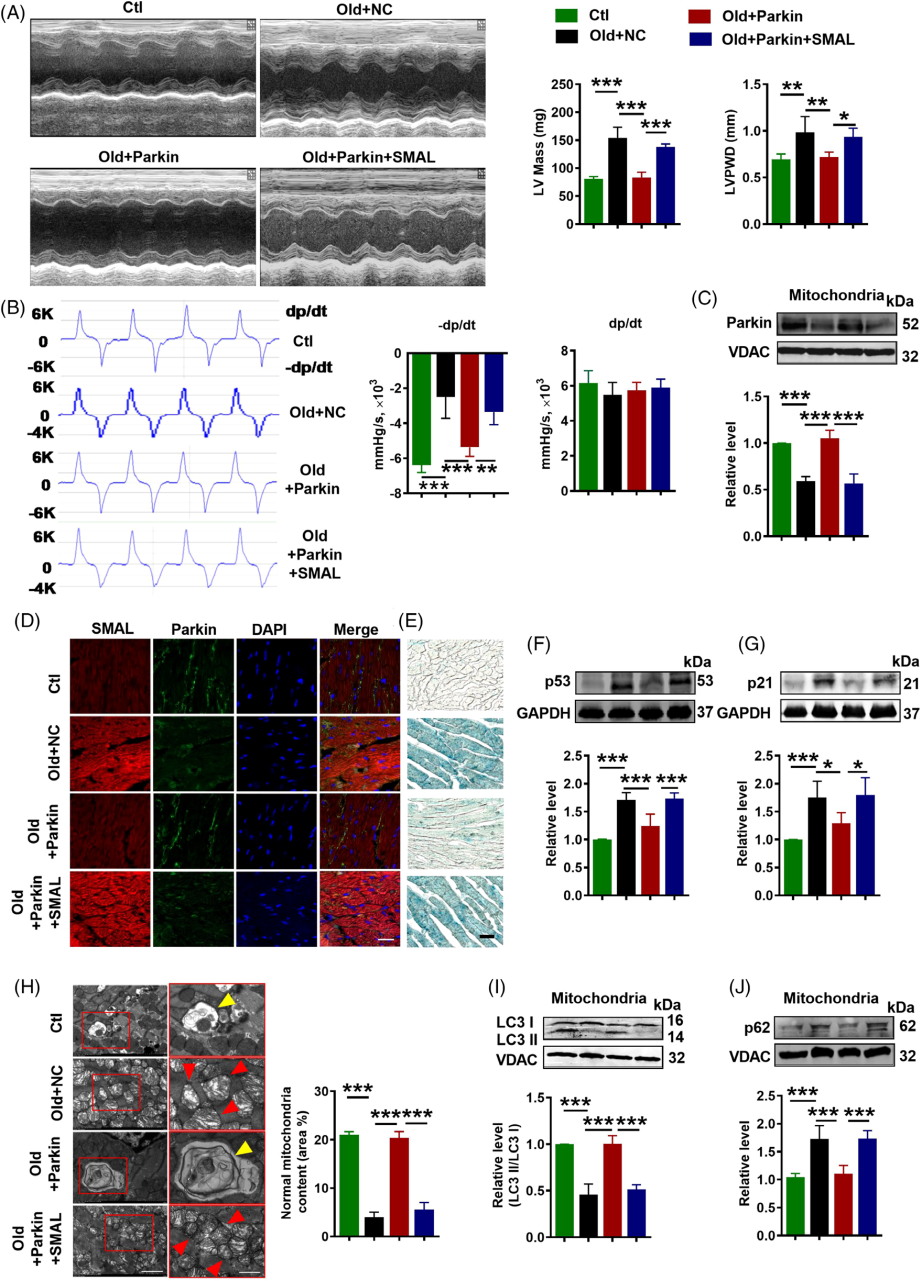
LncR‐SMAL reverses Parkin‐mediated improvement of cardiac function in aged mice. (A) Left ventricular (LV) mass and left ventricular posterior wall thickness (LVPWD) measured by echocardiography; *n* = 5. (B) *±dp*/*dt*_max_ measured by pressure–volume measurements system; *n* = 5. (C) Parkin protein level in mitochondria tested by western blot; *n* = 5. (D) Levels of lncR‐SMAL (red) and Parkin (green) verified by fluorescence in situ hybridization (FISH) and immunofluorescence staining, nuclei (blue). Scale bar: 20 μm; *n* = 5. (E) Cardiac senescence tested by β‐galactosidase staining. Scale bar: 5 μm; *n* = 5. (F and G) p53 and p21 protein levels tested by western blot; *n* = 5. (H) Autophagy examined by an electron microscope. Yellow arrow represents mitophagy vacuole; red arrow represents damaged mitochondria. Scale bar: 2 and 1 μm; *n* = 3. (I and J) light chain 3 (LC3) and p62 protein levels in mitochondria tested by western blot; *n* = 5. *p < .05, **p < .01, ***p < .001; the data were expressed as the mean ± SD.

The previous results suggest that Parkin is a downstream component of lncR‐SMAL, and it might mediate the effect of the latter on heart ageing. To examine this notion, we turned to look at the effects of AAV9‐Parkin infection in 21‐month aged mice (old + Parkin), together with co‐infection of AAV9‐SMAL (old + Parkin + SMAL). Cardiac function and ageing progress were measured 3 months after plasmid transfection. The results showed that Parkin overexpression significantly improved the defective diastolic function of aged mice, as indicated by the decreased LV Mass and LVPWD (Figure 5A), as well as the improved −*dp*/*dt*_max_ (Figure 5B). +*dp*/*dt*_max_, EF and FS remained unaltered in aged mice relative to the young littermates (Figure S6A), and overexpression of Parkin did not significantly affect these parameters. Moreover, Parkin overexpression remarkably inhibited SASP production in ageing mice, whereas the phenomenon was completely blocked by lncR‐SMAL (Figure S6B). Parkin protein level was detected, as shown in the western blot and immunofluorescence staining results. Remarkable reduction in Parkin protein levels was shown in total cell lysate and mitochondrial content of ageing heart and lncR‐SMAL effectively decreased Parkin protein level even in the presence of Parkin overexpression plasmid (Figure 5C,D) (Figure S6C). Moreover, β‐gal staining and western blot suggested that increased Parkin remarkably inhibited cardiac senescence, which was reversed by lncR‐SMAL (Figure 5E–G). TEM examination revealed a remarkable increase in mitophagy and healthy mitochondria content in ageing heart by Parkin overexpression (Figure 5H). However, lncR‐SMAL abrogated the mitophagy induced by Parkin with downregulation of LC3 II/I, PINK1 and BNIP3 and elevation of autophagosome adaptor protein p62 (Figure 5I,J) (Figure S6D–G). To assess the effects of lncR‐SMAL on mitophagy and senescence in Parkin null cells, CRISPR/Cas9 plasmid was transfected into AC16 cells to knockout Parkin expression (Parkin‐KO). The results showed that Parkin‐KO remarkably inhibited cell mitophagy, as shown by the decreased Parkin and LC3 II/I protein levels and increased p62 protein levels in western blot results (Figure S7A–C). Immunofluorescence staining suggested Parkin‐KO exhibited a decrease in both autophagosome and autolysosome formation (Figure S7F). Mitochondria‐targeted Mito‐Keima plasmid (Mito‐Keima) was used to monitor mitophagy. The results showed that Keima dots (red, mitophagy‐autolysosomes) were strongly diminished in Parkin‐KO cells (Figure S7G). As anticipated, Parkin‐KO induced cell senescence as indicated by significant increasing of p53 and p21 protein levels (Figure S7D,E). However, si‐SMAL loses the strong effects of amelioration of mitophagy and senescence after Parkin‐KO (Figure S7A–G). These results further confirmed the regulatory relationship between lncR‐SMAL and Parkin in mitophagy and senescence.

### 2.6 d‐Galactose induces senescence‐like cell phenotypes

**FIGURE 6:**
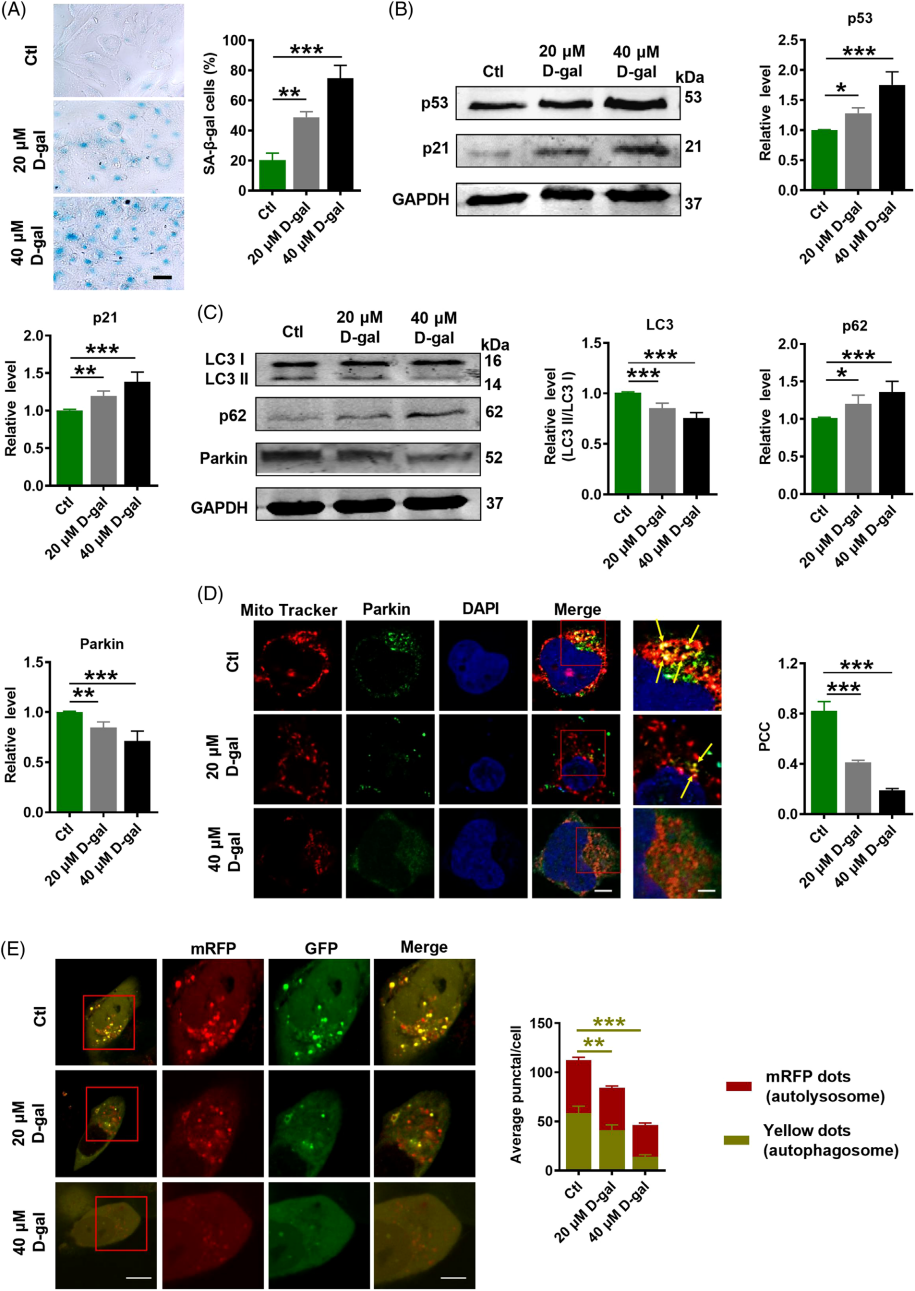
d‐Galactose induces senescence‐like cell phenotypes. (A) Representative images of β‐galactosidase staining and statistical results of positive cells. Scale bar: 200 μm; *n* = 3. (B and C) p53, p21, light chain 3 (LC3), p62 and Parkin protein levels tested by western blot; *n* = 5. *p < .05, **p < .01, ***p < .001 versus Ctl. (D) The subcellular co‐localization (yellow arrows) of Parkin protein (green) and mitochondria (Mito Tracker signal, red) verified by immunofluorescence staining. Scale bar: 20 and 10 μm; co‐localization calculated using Pearson's correlation coefficient (PCC); *n* = 3. (E) Autophagosomes (yellow dots) and autolysosomes (red dots) measured by immunofluorescence staining. Scale bar: 20 and 10 μm; *n* = 3. **p < .01, ***p < .001 versus Ctl. The data are expressed as the mean ± SD.

To verify the suppressive effects of lncR‐SMAL on Parkin‐mediated mitophagy in cardiac senescence, we used d‐galactose (d‐gal; 20 and 40 μM) to induce cell senescence in AC16 cells. Consistently, d‐gal induced remarkable senescence phenotypes, including higher SA‐β‐gal activity and upregulation of p21 and p53 protein levels (Figure 6A,B). Moreover, LC3 II/I and Parkin protein levels were downregulated in response to d‐gal treatment. Increased p62 protein level also suggested the suppressive mitophagy by d‐gal (Figure 6C). Immunofluorescence staining revealed that the localization of Parkin protein to mitochondria was reduced after d‐gal treatment (Figure 6D). Additionally, tandem RFP‐GFP‐LC3 adenovirus was transfected into AC16 cells, and immunofluorescence staining demonstrated that d‐gal diminished yellow (autophagosome) and red (autolysosome) fluorescent protein signals indicating that d‐gal suppressed autophagosome and autolysosome formation (Figure 6E).

### 2.7 Knockdown of lncR‐SMAL prevents cardiomyocytes senescence

**FIGURE 7:**
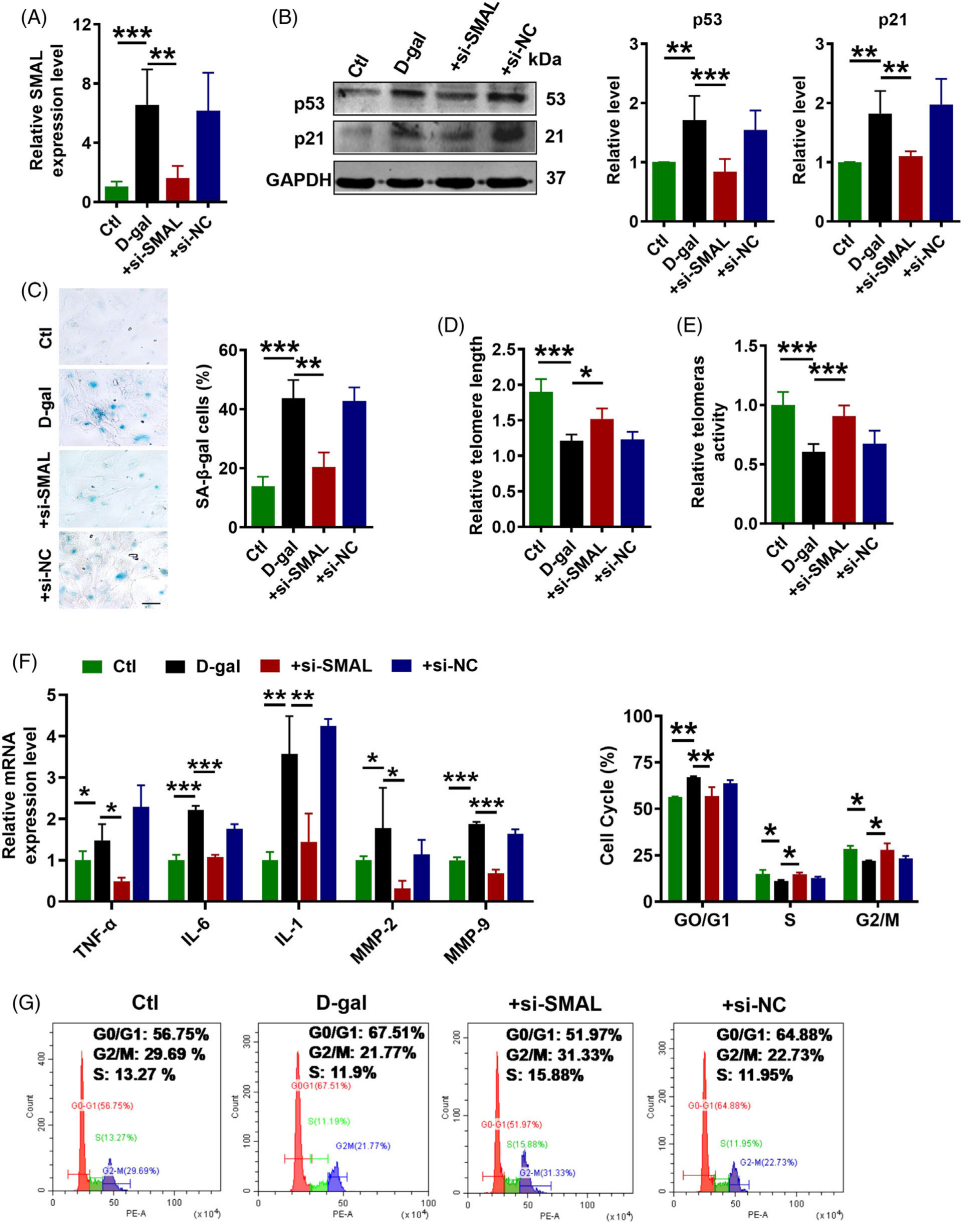
Knockdown of lncR‐SMAL by siRNA (si‐SMAL) delays cardiomyocyte senescence and improves mitophagy. (A) lncR‐SMAL levels tested by quantitative real‐time PCR (qRT‐PCR); *n* = 5. (B) The protein levels of p53 and p21 tested by western blot; *n* = 5. (C) Representative images of β‐galactosidase staining and statistical results of positive cells. Scale bar: 200 μm; *n* = 3. (D) Telomere length detected by qRT‐PCR with a Biowing Telomere Detection Kit; *n* = 5. (E) Telomerase activity detected by qRT‐PCR with a Human Telomerase Activity SYBR Green Real‐time qPCR Kit; *n* = 5. (F) Senescence‐associated secretory phenotype (SASP) TNF‐α, IL‐6, IL‐1, MMP‐2 and MMP‐9 mRNA levels tested by qRT‐PCR; *n* = 3. (G) Cell cycle distribution measured by flow cytometry and calculated in different phases of cell cycle; *n* = 3. *p < .05, **p < .01, ***p < .001; the data were expressed as the mean ± SD.

We reasoned that if lncR‐SMAL indeed played a role in cardiomyocyte senescence, then knockdown of this lncRNA should be able to retard senescence process. To test this thought, we transfected lncR‐SMAL siRNA (+si‐SMAL) into AC16 cells, and 6 h later, d‐gal (40 μM) was added to induce senescence. The efficacy of si‐SMAL to suppress the upregulated lncR‐SMAL level in the d‐gal group was first verified (Figure 7A). Then, a series of senescence‐associated phenotypes were tested. Western blot results showed that si‐SMAL decreased p21 and p53 protein levels in senescent AC16 cells (Figure 7B). β‐Gal staining showed that si‐SMAL reversed d‐gal induced cell senescence (Figure 7C). Telomere length and telomere activity are biomarkers of senescent cells. We also found si‐SMAL significantly extended telomere length and increased telomerase activity (Figure 7D,E). In addition, SASP gene expression was significantly inhibited by the knockdown of lncR‐SMAL (Figure 7F). Flow cytometry analysis of cell cycle indicated that the majority of cells were arrested in the G0/G1 phase, accompanied by a decrease in proportion of cells in the S or G2/M phase in the d‐gal‐treated group, which was reversed by si‐SMAL (Figure 7G). Moreover, to further prove the connection between mitophagy and senescence, BAFA1 was used to block autophagosome‐lysosome fusion in senescent cardiomyocytes. Our data suggested that after pre‐administration of BAFA1, the inhibitory effects of si‐SMAL on senescence were significantly diminished, as indicated by the upregulation of p53 and p21 expression, as well as increasing in SA‐β‐gal activity (Figure S8A–C). The previous results demonstrated that knockdown of lncR‐SMAL could effectively attenuate cardiomyocytes senescence.

### 2.8 Knockdown of lncR‐SMAL promotes cardiomyocytes mitophagy and maintains mitochondrial quality control

**FIGURE 8:**
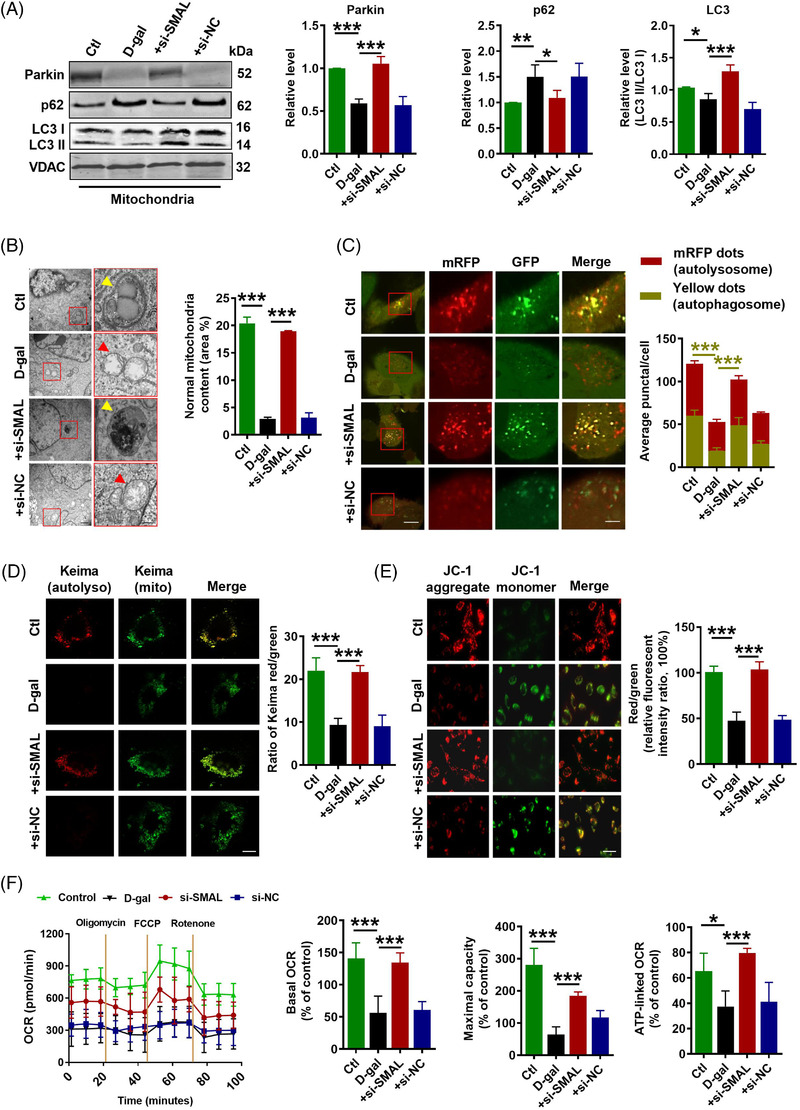
LncR‐SMAL affects mitochondrial function by regulating mitophagy. (A) The protein levels of Parkin, p62 and light chain 3 (LC3) in mitochondria tested by western blot; *n* = 5. (B) Autophagy examined by an electron microscope. Yellow arrow represents mitophagy vacuole; red arrow represents damaged mitochondria. Scale bar: 1 μm and 500 nm; *n* = 3. (C) Autophagosomes (yellow dots) and autolysosomes (red dots) measured by immunofluorescence staining. Scale bar: 20 and 10 μm; *n* = 3. (D) Mito‐Keima fluorescence detected and Mito‐Keima fluorescence intensity ratio (red: 550 nm/green: 440 nm) calculated. Scale bar: 20 μm; *n* = 3. (E) JC‐1 fluorescence detected and JC‐1 fluorescence intensity ratio (red: 590 nm/green: 530 nm) calculated. Scale bar: 0 μm; *n* = 3. (F) Oxygen consumption rates (OCR) measured by Seahorse XFe24 Analyzer; *n* = 5. *p < .05, **p < .01, ***p < .001, the data were expressed as the mean ± SD.

On the other hand, cell mitophagy and mitochondrial function were evaluated after si‐SMAL treatment. Elevated protein levels of Parkin and LC3 II/I, but diminished p62 protein levels in both total cell lysate and mitochondrial content were observed in si‐SMAL group (Figure 8A) (Figure S9A). In addition, the expressions of mitophagy‐related markers PINK1 and BNIP3 were also increased after si‐SMAL treatment (Figure S9B,C). Consistent with previous results, Parkin mRNA level was not influenced by lncR‐SMAL knockdown (Figure S9D). Remarkably, BAFA1 treatment suppressed the promotion effects of si‐SMAL on autophagy, which was shown by the accumulation of LC3 II and p62 proteins within cells, as well as blocked autophagic flux (Figure S8D–F). TEM (Figure 8B) and immunofluorescence staining (Figure 8C) suggested that autophagosome and autolysosome were remarkably accumulated after si‐SMAL treatment; meanwhile, statistical analysis based on TEM images suggested that mitochondria content was also increased by si‐SMAL. Moreover, Mito‐Keima showed that Keima dots (red, mitophagy‐autolysosomes) were reduced in senescent cardiomyocytes, which were also increased by si‐SMAL (Figure 8D). Mitochondrial membrane potential (MMP) was determined with JC‐1 staining, which was normally decreased in senescent cells.^37^ As shown in Figure 8E, the red/green fluorescence intensity ratio was decreased obviously after the cells were incubated with d‐gal, demonstrating that the d‐gal ‐induced oxidative stress greatly lowered MMP of AC16 cells. The treatment of si‐SMAL could cause a larger enhancement of red/green fluorescence intensity ratio, revealing the strong ability of si‐SMAL to stabilize the MMP. To evaluate the mitochondrial respiratory capacity, we measured the oxygen consumption rate (OCR). Our data showed that the basal respiration, maximal respiration and ATP production of d‐gal‐treated cells were significantly lower than that in control cells, whereas knockdown of lncR‐SMAL remarkably ameliorated these impairments (Figure 8F). These results demonstrated that knockdown of lncR‐SMAL could ameliorate mitophagy and mitochondrial dysfunction in senescent AC16 cells.

## 3 DISCUSSION

The aims of this study were to elucidate the pathophysiological role of lncR‐SMAL and the underlying mechanisms in heart ageing. Our data demonstrated that the levels of lncR‐SMAL were robustly increased in serum samples of human subjects over 60‐year old relative to young controls, and in both nucleus and cytoplasm of senescent AC16 cells. Importantly, such an upregulation produced significant senescence‐promoting effects on the heart. This was supported by the findings that artificial overexpression of lncR‐SMAL in otherwise normal mice created a similar impairment of cardiac function as that observed in naturally aged mice. Moreover, lncR‐SMAL diminished the anti‐heart ageing and mitophagy promoting effects of Parkin overexpression in natural ageing mice. At the cellular level, lncR‐SMAL overexpression facilitated the cardiomyocytes senescence; in sharp contrast, knockdown of endogenous lncR‐SMAL in cardiomyocytes partially abrogated the d‐gal ‐induced cell senescence, mitophagy impairment and mitochondrial dysfunction. Moreover, the beneficial effects of si‐SMAL on mitophagy and senescence were largely diminished after Parkin‐KO and BAFA1 treatment. At the molecular level, lncR‐SMAL was found to directly bind to Parkin protein and promote its ubiquitination, which resulted in Parkin protein instability and reduction in functional level during cardiomyocytes senescence. These findings allowed us to propose the following paradigm for the regulation of mitophagy by lncR‐SMAL in the setting of heart ageing: heart ageing →lncR‐SMAL↑ →Parkin↓ →mitophagy↓ →cell senescence↑ →cardiac function↓, as schematically illustrated in *Central Illustration*. Based on these findings, we concluded that lncR‐SMAL is a detrimental lncRNA contributing to cardiac dysfunction through targeting Parkin and the associated mitophagy in ageing heart.

Heart ageing is characterized by unfavourable cardiac remodelling and diastolic function.^38^, ^39^, ^40^, ^41^ Lehallier et al. recently have reported that the number of differentially expressed proteins in human plasma at each age uncovered three crests at ages 34, 60 and 78, and the three crests are largely composed of different proteins.^42^ In the present study, the marked serum crest for heart ageing indicated by overexpressed lncR‐SMAL level was at 60‐year old. Meanwhile, lncR‐SMAL did not present continuous upregulation as age increased. However, whether serum lncR‐SMAL level correlated with parameters of diastolic dysfunction in ageing population was not capable of being concluded based on current study, large‐scale clinical studies would be valuable to answer the question and further evaluate the clinical use of lncR‐SMAL. Therefore, these studies together with us confirmed that age 60 was an important time point for heart ageing progression.

Mitophagy maintains physiological homeostasis in all kinds of cells within body, due to its role in regulation of oxidative stress and energy metabolism.^43^ Defective mitophagy has been described in multiple ageing threatening diseases, including neurodegenerative disorders,^44^ atherogenesis,^29^ heart failure^26^, ^45^ and cardiac arrhythmias.^46^ In cardiomyocytes, the less dividing nature determines that they are highly dependent on mitophagy for the renewal of non‐functional mitochondria, proteins and other organelles. There is increasing evidence that the activation of mitophagy in cardiomyocytes is an adaptive response to stimulation, and inhibition of mitophagy accelerates cardiac ischaemia‐reperfusion injury.^47^ We found here that the key mitophagy regulator protein Parkin was dysregulated in heart ageing. Tyrrell et al. have reported the increased level of Parkin in the aortas of aged mice (18‐month mice).^29^ Hoshino et al. do not observe any changes of Parkin level in the whole cell lysate of cardiomyocytes from 18‐month mice.^28^ In another study, Parkin was found downregulated in the heart of aged mice (24‐month mice).^36^ These studies demonstrated that the role of Parkin in ageing process was age related and with tissue differences. Despite several insights gained into the dysregulation of Parkin‐directed mitophagy in heart ageing, truly regulation on disease‐relevant phenotypes and upstream triggers remain scarce. We first tested the Parkin protein level in heart from different age of mice. The results confirmed a consistent time‐point characteristic with previously published studies for Parkin expression during heart ageing, as indicated by a sharp decrease in a certain time point (21‐month mice). The beneficial roles of Parkin in preventing heart ageing in natural ageing mice were proved by our data. Moreover, the impairment of mitophagy caused by Parkin‐KO and BAFA‐1 was demonstrated in AC16 cells, which resulted in significant cardiomyocytes senescence. Consistently, our previous published study and others also revealed that upregulation of Parkin could ameliorate heart ageing or cardiomyocytes senescence. We found emodin and its derivative could ameliorate heart ageing by stabilizing Parkin protein.^48^ Hoshino et al. reported that Parkin‐mediated mitophagy was impaired in the hearts of aged (20‐month old) mice compared to young (10‐month old) mice.^28^ Manzella et al. Proved that the inhibition of Parkin‐mediated mitophagy by monoamine oxidase‐A is a novel driver of premature senescence in cardiomyocytes.^49^ The consistent conclusion was also summarized in some reviews.^50^, ^51^ Hence, these results reinforce the evidence for a direct connection between mitophagy and senescence.

When overexpression of lncR‐SMAL by fourfold to a similar level as that increased in natural ageing heart, the anti‐heart ageing effects of Parkin were strongly diminished and heart ageing phenotypes were also induced in young mice. A similar phenomenon was also observed in AC16 cells and neonatal mouse primary cardiomyocytes. Therefore, we concluded that lncR‐SMAL had a strong heart ageing induction effect. The anti‐senescence effects of lncR‐SMAL knockdown were detected in vitro. Si‐SMAL was able to rescue the deleterious actions of d‐gal on cell senescence. Moreover, using BAFA‐1 and Parkin‐KO to inhibit mitophagy, the important role of si‐SMAL‐mediated anti‐senescence effects was also demonstrated. Mitophagy deficiency was able to induce mitochondria dysfunction. As shown in several published studies, decreased mitophagy could induce the accumulation of abnormal mitochondria, which resulted in oxidative stress and further decreased MMP, impaired OCR.^52^, ^53^ Consistently, our data also suggested that increasing of mitophagy by si‐SMAL effectively ameliorated mitochondrial dysfunction, including mitochondria content loss, MMP and OCR impairment. Besides, mitophagy‐related genes such as PINK1 and BNIP3 were also influenced by lncR‐SMAL. We have predicted that there was no direct interaction between PINK1 and lncR‐SMAL. It might be possible that the altered expression of PINK1 and BNIP3 was due to the alteration of Parkin expression. The phenomenon had also been proved in several published studies.^54^, ^55^ However, there might be other regulatory mechanisms between lncR‐SMAL and PINK1 or BNIP3, which was valuable to investigate in subsequent studies.

Large amount of evidence has revealed the regulatory mechanisms of lncRNA on functional proteins.^56^, ^57^, ^58^ Our previous studies have reported that lncRNAs, such as lncR‐ZFAS1, lncR‐CCRR and lnR‐MIAT, could inhibit the protein function through directly binding to its functional region.^13^, ^16^, ^17^ Consistent with the previous studies, here we found that lncR‐SMAL reduced Parkin protein levels both in whole cell lysate and mitochondrial content and restricted the mito‐translocation of Parkin. Notably, lncR‐SMAL interacted with Parkin protein indicated by computational docking analysis, RIP and pull‐down assays. Parkin protein instability caused by lncR‐SMAL is possible resulted from the changes of protein conformation, which further induced the endogenous degradation of Parkin protein through ubiquitination, and the analogous molecular mechanism was also reported in other studies.^59^, ^60^, ^61^ Nonetheless, these facts together with our findings would suggest that the direct interaction between lncRNAs and protein functionally contributes to protein reduction and subsequent molecular process.

Our findings suggest that lncR‐SMAL seems to play an essential role in the regulation of cardiac geometry and function in ageing. The expression crest of lncR‐SMAL in serum might provide a reference for human heart ageing. Our data favour the notion that increased lncR‐SMAL with advanced ageing may underscore reduced mitophagy in ageing, indicating the therapeutic potentials for lncR‐SMAL and mitophagy in the management of heart ageing.

### 3.1 Possible limitations of our study

Here we revealed that the upregulation of lncR‐SMAL contributes to the defective mitophagy and increased heart ageing. However, further studies are required to clarify how lncR‐SMAL is upregulated with increasing age and explore pharmacological tools to block this pathway. The early warning biomarkers for ageing, especially for ageing heart, are rare. Although we have found that lncR‐SMAL is increased in serum of people over 60‐year old, more detail and related factors need to be involved for further evaluating the warning effects of blood lncR‐SMAL levels on heart ageing. Moreover, we proved the transcription of lncR‐SMAL from mouse genome; nevertheless, the whole sequence of mmu‐lncR‐SMAL still needs further identification.

## 4 MATERIALS AND METHODS

### 4.1 Human blood samples

Twenty‐five young and old people who received physical examination at the Second Affiliated Hospital of Harbin Medical University from February 2017 to March 2018 were selected. Serum from people of different ages was collected and stored until use. Participants were excluded according to the following criteria: hypertension (SBP ≥ 140 mmHg or DBP ≥ 90 mmHg), or taking antihypertensive medication; diabetes mellitus with fasting glucose ≥126 mg/dl (7.0 mmol/L) or using hypoglycemic agents; cardio‐cerebrovascular disease (coronary artery disease, myocardial infarction, ischemic stroke, heart arrhythmia, cerebral haemorrhage or heart failure with LV EF < 50%); overweight and obese (body mass index ≥25 kg/m^2^); chronic obstructive pulmonary disease with the ratio of forced expiratory volume in the first second to a forced vital capacity of <.7. The Ethics Committees of Harbin Medical University approved study protocols, which were performed in accordance with the ethical principles in the Declaration of Helsinki. Written informed consent was signed by the patient prior to participation. Clinical patient information of human blood samples was shown in Table S1.

### 4.2 Animal care

C57BL/6 female mice with 2 months in age and weighing between 22 and 25 g each were provided by Beijing Vital River Laboratory Animal Technology Co. Ltd. Use of animals was approved by the Ethic Committees of Harbin Medical University and conformed to the Guide for the Care and Use of Laboratory Animals published by the US National Institutes of Health (NIH Publication No. 85‐23, revised 1996).

### 4.3 Establishment of heart ageing model and study design

Female C57BL/6 mice aged 3 and 21 months were housed in a standard animal chamber with a temperature of 22 ± 1°C and a humidity of 55% ± 5%. Animals were randomly divided into Ctl (3‐month old), SMAL (infection of 3‐month‐old mice with AAV‐SMAL once per month for 3 months), NC (infection of 3‐month‐old mice with AAV‐NC per month for 3 months), old + NC (infection of 21‐month‐old mice with AAV9‐NC once per month for 3 months), old + Parkin (infection of 21‐month‐old mice with AAV9‐Parkin once per month for 3 months) and old + Parkin + SMAL (co‐infecting AAV9‐Parkin and AAV9‐SMAL into 21‐month‐old mice once per month for 3 months).

### 4.4 Construction of viral vectors for lncR‐SMAL and Parkin overexpression

AAV has been recognized and widely used for its high infectivity, extensive tissue transduction, low immunogenicity, non‐pathogenicity and long‐term gene expression duration. AAV9 is the most effective of all serotypes for myocardial transduction. AAV9 vector carrying lncR‐SMAL sequence (XR_943203) for overexpression of lncR‐SMAL (AAV9‐SMAL) and a CMV promoter conjugated was constructed (Genechem Co., Ltd., Shanghai, China). AAV9 vector carrying Parkin mRNA sequence (NM_016694) for overexpression of Parkin (AAV9‐Parkin) and a CMV promoter conjugated was constructed (Genechem Co., Ltd., Shanghai, China). Mice in control group and negative control (NC) group were injected with saline and empty vector of AAV9 (AAV9‐NC) at the same dose of AAV via tail vein, respectively.

### 4.5 In vivo gene delivery

C57BL/6 mice were randomized to receive the virus solution (1 × 10^11^ genome containing particles /animal/month; AAV9‐SMAL or AAV9‐Parkin or AAV9‐NC constructs diluted to 100 μl saline) and administrated via tail vein injection. For data collection following single‐gene infection, experimental measurements were carried out 3 months following in vivo injection of AAV9‐SMAL for lncR‐SMAL overexpression or AAV9‐Parkin for Parkin overexpression. For acquiring data on co‐infection of AAV9‐SMAL and AAV9‐Parkin, experimental measurements were taken after the 3 months of AAV9‐Parkin and AAV9‐SMAL injection.

### 4.6 Echocardiographic assessment of cardiac function

Mice were anaesthetized with .2‐g/kg avertin intraperitoneally (Sigma, St Louis, MO, USA). LV function was assessed by an ultrasound machine Vevo2100 high‐resolution imaging system (VisualSonics, Toronto, ON, Canada). Mice were placed on either their back or their side on a warming mat with their body temperature being maintained at 37°C. End‐diastolic and end‐systolic areas were measured according to the American Society of Echocardiography leading edge method. LVPWD was detected, and LV mass, EF and FS were calculated from M‐mode recording.

### 4.7 Measurements of cardiac contractility

Cardiac contractility was assessed with an ADVANTAGE pressure‐–volume measurements system (#FY897B; Science, London, England). In brief, mice were anaesthetized. Systolic, diastolic, LV pressure, and the peak and minimum values of ±*dp*/*dt*_max_ were measured by a catheter passing through the right carotid artery into the left ventricle. Haemodynamic parameters were recorded by LabScribe software (Iworx, Dover, DE, USA) after 10 min of stabilization.

### 4.8 Culturing of AC16 cell line

The AC16 cell line (#SCC109, Millipore Sigma, MMAS, USA), which is derived from humans and exhibits a variety of morphological and biochemical characteristics of cardiomyocytes, was cultured in the manner described previously. Briefly, non‐differentiated AC16 cells were cultured in a DMEM:F12 medium (#SH30023.01; HyClone, Logan, UT, USA), which has added 10% fetal bovine serum (FBS), 1% penicillin‐streptomycin and 1% fungizone (#C0222; Beyotime, Shanghai, China), and grown in a cell incubator (5% CO_2_, 37°C). For transfection procedures, the AC16 cells were starved in DMEM without serum for 24 h and then transfected with 100‐nM lncR‐SMAL siRNA (si‐SMAL) (RiboBio Co., Ltd., Guangzhou, Guangdong, China) using an X‐treme RNA transfection reagent (Invitrogen, Ltd., America); meanwhile, a non‐sense scramble sequence of si‐SMAL was used as NC (si‐NC). The transfection mixture was dissolved in Opti‐MEM (Gibco, NY, USA) (10%) and DMEM without serum (90%), finally added to the cells. LncR‐SMAL‐overexpressing pcDNA3.1‐plasmid (100 nM) was transfected into AC16 cells using a Lipofectamine 2000 reagent (Invitrogen, Carlsbad, CA, USA) as specified and recommended in the reagent instructions. After 6 h, the medium was replaced by fresh medium added d‐gal (20 μM or 40 μM; Sigma‐Aldrich, St. Louis, MO, USA) or not. After 48 h, follow‐up experiments were carried out. For Parkin CRISPR/Cas9 KO plasmid transfection, a 3‐μg Parkin CRISPR/Cas9 KO Plasmid and a 15‐μl UltraCruz Transfection Reagent (#sc‐395739, Santa Cruz Biotechnology, CA, USA) were diluted into a 100‐μl plasmid transfection medium (#sc‐108062, Santa Cruz Biotechnology, CA, USA), mixed well and left to rest for 5 min, respectively. The two were evenly mixed and left to stand for 20 min. Finally, a 200‐μl complex was added to the prepared six‐well plate AC16 cells (2 ml medium in total). At 48 h after transfection, follow‐up experiments were conducted.

### 4.9 Culturing of human umbilical vein endothelial cells (HUVECs) and RAW264.7

Human umbilical vein endothelial cells (HUVECs) (CTCC‐0804‐PC, Meisen, Zhejiang, China) and mouse RAW264.7 (ZQ0098, Zhongqiaoxinzhou Biotech, Shanghai, China) were cultured as previously described.^62^, ^63^ Briefly, HUVECs and RAW264.7 were cultured in an RPMI 1640 medium (#8121486; Thermo Fisher Scientific, Beijing, China) and DMEM:F12 medium, supplemented with FBS (10%), penicillin‐streptomycin (1%), respectively. Cells were maintained in a cell incubator (5% CO_2_, 37°C). After 48 h, cells were harvested for RNA extraction.

### 4.10 Primary culturing of neonatal mouse ventricular cells (NMVCs) and neonatal mouse fibroblasts (NMFs)

Mice ventricular tissues from neonatal mice (1–3 day) were trypsinized by .25% trypsin at 4°C for 10 h and then dissociated in Collagenase II (.8 mg/ml) (#17101015; Gibco, NY, USA) dissolved with DMEM. Heart tissues were digested until there was no visible tissue left. Cells collected by centrifugation (1500 rpm/min, 5 min) were resuspended in DMEM and added with FBS and penicillin‐streptomycin same as before. After cells were cultured in an incubator (5% CO_2_, 37°C) for 1.5 h, the ventricular cell suspension was cultured with DMEM‐containing arabinoside (10 nM) to suppress cardiac fibroblasts. The adherent‐grown fibroblasts were continued in the primary growth environment with fresh DMEM.

### 4.11 Senescence‐associated β‐galactosidase staining (SA‐β‐gal)

A senescence β‐galactosidase staining kit (Cell Signaling Technology, Boston, USA) was used as specified and recommended in the kit instructions. Briefly, cells or frozen sections of heart were fixed (room temperature, 10–15 min) and washed twice with phosphate‐buffered saline (PBS). Next, cells or frozen sections were added with 1 ml β‐galactosidase staining solution and placed in a dry incubator (no CO_2_) at 37°C for 12 h, observed by a light microscope (Carl Zeiss Microscopy, Jena, Germany).

### 4.12 RNA‐interacting protein immunoprecipitation (RIP)

The Magna RIP RNA‐Binding Protein Immunoprecipitation Kit (Cat#17‐701; Millipore, Darmstadt, Germany) was purchased to complete RIP assay as specified and recommended in the kit instructions. The ReverTraAce qPCR RT Kit (#FSQ‐101; Toyobo, Osaka, Japan) was used to reverse transcription RNA into cDNA. The SYBR Green PCR Master Mix Kit (Cat#4309155; Invitrogen) was used for qRT‐PCR of the target genes using the LightCycler 96 Real‐Time PCR System (Roche, Basel, Switzerland). Briefly, reactions were done in 20‐μl volumes (1‐μl each primer; 1‐μl cDNA; 10‐μl Power SYBR Green PCR Master Mix; 7‐μl nuclease‐free water) and completed with cycling parameters recommended by manufacturers. The 2^−ΔΔ^ *^CT^* method was carried out to assess relative expression levels. The melting curve was analysed to determine the specificity/quality of amplification.

### 4.13 RNA‐protein pulldown

LncR‐SMAL (homo sapien) plasmid was constructed into a pcDNA3.1 vector (Invitrogen). The T7 RNA Polymerase Kit (Cat#D7069; Beyotime, Shanghai, China) was used for RNA transcription as specified and recommended in the kit instructions for in vitro experiments. The transcripts were labelled by RNA 3′ End Desthiobiotinylation Kit (Cat#20163; Oshkosh, WI, USA) as specified and recommended in the kit instructions. The Pierce Magnetic RNA‐Protein Pull‐Down Kit (Cat#20164; Pierce) was used in RNA‐protein pulldown experiments as specified and recommended in the kit instructions. The protein pulled down by LncR‐SMAL (Parkin) was detected by western blot.

### 4.14 Co‐immunoprecipitation (CO‐IP) assay and ubiquitination analysis

After 12 h MG‐132 (#2194; CST, Danvers, Massachusetts, USA) treated, the cells were washed with pre‐cooled PBS and lysed with cold RIPA buffer. The protein concentrations were measured by a BCA Protein Assay kit (Bio‐Rad, Mississauga, ON, Canada). And 1‐mg protein lysate was combined with 60‐μl Protein A/GPLUS‐Agarose (Santa Cruz Biotechnology, Shanghai, China) pre‐incubated with mouse anti‐Parkin antibody followed by incubation (4°C, ≥12 h) by vertical shaking. Cell lysates were centrifuged (5000 rpm/min, 5 min), and then obtained precipitation was sequentially washed three times with TBST buffer. Immunoprecipitate was adding RIPA and 6× loading buffer then heating at 100°C for 8 min followed by western blot analysis. Input samples were divided from the same treatment, and directly added RIPA and 6× loading buffer then heated. Immunoprecipitate samples performed western blot with rabbit anti‐ubiquitin antibody (#58395, Cell Signaling Technology, MA, USA), and input samples performed with mouse anti‐Parkin antibody (#4211, Cell Signaling Technology, MA, USA).

### 4.15 Electron microscopic examination

After being fixed with 2.5% glutaraldehyde at 4°C overnight, heart tissue and cardiomyocytes were immersed in 2% osmium tetroxide. Next, the prepared sections were dehydrated and stained with uranyl acetate and lead citrate. A JEOL 1200 electron microscope (JEOL Ltd, Tokyo, Japan) was used to observe the changes of cardiomyocytes’ microstructure.

### 4.16 Tissue handling and immunohistochemistry assessment

After being fixed with 10% formalin solution, the heart samples were dehydrated, processed and embedded in paraffin using tissue‐processing equipment. H&E and Masson staining of sections were performed to observe the changes of heart structure.

### 4.17 Flow cytometry

AC16 cells were digested, collected, washed with PBS for three times, centrifuged into clumps (1000 *g*, 5 min) and fixed with 70% pre‐cooled alcohol at 4°C for more than 12 h. The next day, cells washed twice with pre‐cooled PBS were incubated with RNase and propidium iodide (Invitrogen, Carlsbad, CA, USA) (4°C, 30 min) away from light. Cell cycle was measured by flow cytometry CytoFLEX (Beckman Coulter Commercial Enterprise (China) Co., Ltd, Suzhou, China) upon the detection of propidium iodide signal. The results were analysed by CytExpert 2.0 software (Beckman Coulter Company, CA, USA).

### 4.18 Live‐cell imaging for autophagic flux

The mRFP‐GFP‐LC3 adenoviral particles (HanBio, Shanghai, China) were used to infect cells. After 24 h, images of autophagic flux were acquired with a confocal laser microscope (LSM780; Zeiss Microsystems, Jena, Germany).

### 4.19 Mito‐Keima staining for mitophagy

The Mito‐Keima lentivirus was purchased from Public Protein (#ADY211220, Guangzhou, China). The excitation spectrum of Kemia (a pH‐sensitive fluorescent protein) shifts from 440 to 586 nm (neutral pH to acidic pH), when mitochondria were transported to acidic lysosomes, thereby quantification of mitophagy. Images at emission wavelengths of 440 and 550 nm were captured by a laser scanning confocal microscope. ImageJ software was used to quantify fluorescence intensity.

### 4.20 JC‐1 staining of mitochondrial membrane

JC‐1, an indicator which fluoresces red in cells with high Δ*Ψ*_m_, whereas green in low‐potential mitochondria, was used to detect MMP (Δ*Ψ*_m_). Briefly, cardiomyocytes were incubated with JC‐1 (37°C, 20 min), washed for three times and analysed immediately with laser scanning confocal microscopy at emission wavelengths of 530 and 590 nm. Five random fields for each sample were selected for calculating the red/green ratio by using ImageJ software.

### 4.21 Immunofluorescence and immuno‐FISH

For Parkin immunofluorescence staining coupled with Mito Tracker, cells were stained with a Mito Tracker for 30 min, and then fixed in 4% paraformaldehyde and incubated with a primary antibody (4°C, ≥ 12 h), followed by incubation with a fluorescent probe labelled secondary antibody (Invitrogen) for 1 h. For Parkin immunofluorescence staining coupled with lncRNA‐fluorescence in situ hybridization (immuno‐FISH), tissue sections were fixed in 4% paraformaldehyde. Following immunostaining, a RiboTM Fluorescent in situ Hybridization Kit (RiboBio, Guangzhou, China) was purchased to complete FISH fluorescent probe staining of lncR‐SMAL as specified and recommended in the kit instructions. In brief, samples were blocked in a pre‐hybridization buffer (37°C, 30 min), followed by hybridization in hybridization buffer containing 20‐μM lncRNA FISH Probe Mix room temperature ≥12 h. The samples were washed with .1% Tween‐20 in 4 × SSC, 2 × SSC and PBS for 10 min each. DAPI was used to label the nucleus. Finally, the samples were mounted and imaged on a confocal laser microscope.

### 4.22 Seahorse mitochondrial OCR analysis

A Seahorse XFe24 Analyzer (Agilent, CA, America) was used to detect OCRs. AC16 cells were seeded on a V7 assay plate for 24 h and maintained until ∼90% confluency. The cells were transfected with si‐SMAL and incubated with 40‐μM d‐gal for 48 h. Cells were washed twice by a Seahorse XF Base Medium and then added to 500‐μl same medium and incubated (37°C, 1 h). After basal OCR measurement, cells were treated with a vehicle or oligomycin (Port A; 1.5 mg/ml), FCCP (Port B; .5 μM) and Antimycin A (Port C; .5 μM). Finally, OCR was analysed and measured by a Seahorse XFe24 Analyzer.

### 4.23 qRT‐PCR

A TRIzol reagent (Invitrogen, Carlsbad, USA) was used for total RNA extraction as specified and recommended in instructions. Reverse transcription and amplification of RNA were completed using the same experimental methods and reagents as in RIP assay. Relative expression levels of target lncRNA and mRNA were calculated after correction for the expression endogenous reference (β‐actin or GAPDH) in use of the 2^−ΔΔ^ *^CT^* method. Primers used in this study were listed in the following table:

**Table:** 

**LOC105378097 (lncR‐SMAL, human)**	Forward: AGGCCCGGCATGGTTAAGCG
	Reverse: CCACGAAGCCGAGATGCCGT
**LnR‐SMAL (for RIP in AC16)**	Forward: TCGTGGGAGTTGAGGAGCGTCT
	Reverse: TGGGAATGGGCTGCAAGCAGAA
**LnR‐SMAL (for RIP in HEK293)**	Forward: CTCCAGTGGGCTCGACTATT
	Reverse: GACAGCTAAGTTGTATAGAGTAGTG
**LOC105373170 (human)**	Forward: CTCCTGCAGCCACTGATCTA
	Reverse: AGCAGGAGAAACAGTGACGA
**LOC105378351 (human)**	Forward: TGCCAGAGTGGCCAGAATAA
	Reverse: TGGTCGAAGGTCCAAGAGTC
**LOC105379003 (human)**	Forward: ACCTGCAGATTCATGGGACA
	Reverse: CCCTGGGCATATCCATGCTA
**LINC02248 (human)**	Forward: GGAACTGTCCCTCCTGATCC
	Reverse: TCAGGCAGCTTCCTTACACT
**LINC02208 (human)**	Forward: GTGCCTGTTGAAGCTGGAAA
	Reverse: GGCTAGGGCAGGATTGAGAT
**LINC01479 (human)**	Forward: CAAGTGGAATCAAGCAGCCT
	Reverse: CTGCTTGCCTCTTGCCTTAG
**LOC105369998 (human)**	Forward: ATCCATGTGGTCTGCACCAA
	Reverse: TCCTGGAAGGCAATCACTGT
**LOC105371646 (human)**	Forward: TGTGACAGCACAATCATTTCCA
	Reverse: CCCTGCTGCTTGATCTTTGT
**LOC101929258 (human)**	Forward: GATGCACCAGTGATCAACCC
	Reverse: TCACCAGCTCACCTTTGTCT
**LOC101927888 (human)**	Forward: GCAACAGGTGACTGAAGGTT
	Reverse: GTATCTGCAAAAGAAACTAGGC
**LOC101927404 (human)**	Forward: CTGATGTGCAAGTGGCCAGCG
	Reverse: TGGTGATGGCAACACGGTCCTC
**LOC101927145 (human)**	Forward: ATCCTTCACATTTGCCTGCCCCT
	Reverse: TGCCCATCCTTTGTCCCACCA
**LOC107986043 (human)**	Forward: TGGCTGAGACAAAACCTGGGGT
	Reverse: AGGCATTTCTTCTTGAGCCCACT
**LOC102724319 (human)**	Forward: CAGCTGTGGCACTGGCTACCT
	Reverse: GGTGGCAAAGTACTGCAATGACA
**LOC102723850 (human)**	Forward: ACCTGTCTGAGATCACAAGCAAT
	Reverse: AGACATAAGTGGTAAGTCACTGCAC
**LOC101928359 (human)**	Forward: TGCAGAGCAGCAGAGAGTGCC
	Reverse: TGCTGCAGTGCTTCAGTCCCT
**LINC02517 (human)**	Forward: TGCCCGTCCACAGGGTATGC
	Reverse: GCCAGAGTGTCTGCTGAGCCC
**LOC107986462 (human)**	Forward: GGTGGCCAGGAAATGTCCCAGT
	Reverse: AGCCAAGTACGCAAGGCATGGT
**LOC107986203 (human)**	Forward: GCACTCCGCCACAGCTGACA
	Reverse: TAGACGTGGTGGGTGGCGTT
**LOC105379404 (human)**	Forward: TTTTGCCCAGGTCCATGCCA
	Reverse: GGACTCCTCTAATTTTACCTTGGGA
**LOC105372686 (human)**	Forward: ATGCGCCTGGGCTGGAAGAG
	Reverse: CTGCCGGTGTGGGTTGGTGA
**LOC105370446 (human)**	Forward: GCACTTGGAGCCATCCATGCAA
	Reverse: GCACCAAGCTCTCACGCCCG
**LOC101927072 (human)**	Forward: AGGGCTACCAAGATTATTGAGGGGA
	Reverse: TGCTCACACTCAACACAGGGA
**Parkin (human)**	Forward: CTGGAAGTCCAGCAGGTAGATCA
	Reverse: TACATGGCAGCGGGGACAGG
**GAPDH (human)**	Forward: AAGAAGGTGGTGAAGCAGGC
	Reverse: TCCACCACCCAGTTGCTGTA
**GAPDH (mouse)**	Forward: AGTTCAACGGCACAGTCAAG
	Reverse: TACTCAGCACCAGCATCACC
**β‐actin (human)**	Forward: GGCTGTATTCCCCTCCATCG
	Reverse: CCAGTTGGTAACAATGCCATGT
**Primer 1**	Forward: GAGGCCAGTTTTAGGGGAGT
	Reverse: GCTCAGACAAAGGAATGGGAC
**Primer 2**	Forward: GGCCAGTTTTAGGGGAGTAGT
	Reverse: GGTGCTCAGACAAAGGAATGG
**Primer 3**	Forward: ACGAGGCCAGTTTTAGGGG
	Reverse: GAGGTGCTCAGACAAAGGAA
**TNF‐α (human)**	Forward: TGCTCCTCACCCACACCATCAG
	Reverse: TCCCAAAGTAGACCTGCCCAGAC
**TNF‐α (mouse)**	Forward: CACCACGCTCTTCTGTCTACTGAAC
	Reverse: TGACGGCAGAGAGGAGGTTGAC
**IL‐1 (human)**	Forward: TACGAATCTCCGACCACCACTACAG
	Reverse: ACACCACTTGTTGCTCCATATCCTG
**IL‐1 (mouse)**	Forward: TTCAGGCAGGCAGTATCACTCATTG
	Reverse: TGTCGTTGCTTGGTTCTCCTTGTAC
**IL‐6 (human)**	Forward: GACAGCCACTCACCTCTTCAGAAC
	Reverse: CCAGGCAAGTCTCCTCATTGAATCC
**IL‐6 (mouse)**	Forward: CTTCTTGGGACTGATGCTGGTGAC
	Reverse: CTCTCTGAAGGACTCTGGCTTTGTC
**ICAM‐1 (human)**	Forward: GTCACCTATGGCAACGACTCCTTC
	Reverse: TCACTGTCACCTCGGTCCCTTC
**ICAM‐1 (mouse)**	Forward: CCGCTACCATCACCGTGTATTCG
	Reverse: TTAGAGAACAATGCCAGCCCTTGC
**MMP‐2 (human)**	Forward: CGACCACAGCCAACTACGATGATG
	Reverse: GTGCCAAGGTCAATGTCAGGAGAG
**MMP‐2 (mouse)**	Forward: TGTGTTCTTCGCAGGGAATGAGTAC
	Reverse: CACGACGGCATCCAGGTTATCAG
**MMP‐9 (human)**	Forward: CCTTCCTTATCGCCGACAAGTGG
	Reverse: GTAGAAGCGGTCCTGGCAGAAATAG
**MMP‐9 (mouse)**	Forward: CGCCACCACAGCCAACTATGAC
	Reverse: GATACTGGATGCCGTCTATGTCGTC

### 4.24 DNA extraction and telomere length detection

Total DNA of AC16 cells was extracted using DNAzol LS (Invitrogen, Carlsbad, USA) as specified and recommended in the reagent instructions. And LightCycler 96 Real‐Time PCR System was used to detect telomere length in extracted DNA with a Biowing Telomere Detection Kit (#TL50, Biowing, Shanghai, China).

### 4.25 Telomerase activity detection

Total RNA was extracted from AC16 cells and synthesized into cDNA. Telomerase activity was detected by LightCycler 96 Real‐Time PCR System with a Human Telomerase Activity SYBR Green Real‐time qPCR Kit (#KFS307, Biorab, Beijing, China).

### 4.26 Mitochondrial isolation

Mitochondria protein from AC16 cells and heart tissue were purified using two mitochondria isolation kits (#C3601, C3606; Beyotime, Shanghai, China) under the guidance of the instructions. Briefly, cells or frozen sections of heart were washed with pre‐cooled PBS and then added mitochondrial separation reagent or PMSF. Density gradient centrifugation at 4°C was used according to instructions. After removing supernatant carefully, the sediment at the bottom is isolated mitochondria protein. Then the protein was used for western blot analysis.

### 4.27 Western blot analysis

Protein samples (60–100 μg/well) were attached to nitrocellulose membranes (PALL, New York, USA) by electrophoresis and transfer membrane. Sealed with 5% skim milk, the protein binds to the primary antibodies for p21 (1:500, #sc‐817, Santa Cruz Biotechnology, CA, USA), p53 (1:1000, #25241, Cell Signaling Technology, MA, USA), LC3 (1:500, #12741, Cell Signaling Technology, MA, USA), p62 (1:1000, #5114, Cell Signaling Technology, MA, USA), Parkin (1:500, #sc‐133167, Santa Cruz Biotechnology, CA, USA), PINK1 (1:1000, #A7131, ABclonal, Wuhan, China), BNIP3 (1:1000, #A5683, ABclonal, Wuhan, China), VDAC (1:1000, #4661S, Cell Signaling Technology, MA, USA), GAPDH (1:2000, # TA802519, Clone OTI2D9, OriGene, MD, USA) on a shaker at 4°C overnight. Odyssey Infrared Imaging System (LI‐COR, Lincoln, NE, USA) was used to observe and quantify the experimental results of western blot.

### 4.28 Statistical analysis

The experiments were randomized and blinded. All data were presented as the mean ± SD. Student's *t*‐test was used for statistical comparisons between two groups. One‐way ANOVA with Turkey's multiple comparison test was used to analyse differences among multiple groups (GraphPad Prism 8.3.0). The Kruskal–Wallis H and the Mann–Whitney *U* test were used to analyse data of clinical samples by SPSS 19.0. *p* < .05 was considered statistically significant.

## FUNDING INFORMATION

This study was funded by the National Natural Science Foundation of China (Grant nos.: 91949130, 81961138018, 81903610 and 81730012) and HMU Marshal Initiative Funding (HMUMIF‐21022).

## CONFLICT OF INTERESTS

The authors declare that there is no conflict of interest that could be perceived as prejudicing the impartiality of the research reported.

## Supporting information

Figure S1 LncR‐SMAL is highly specifically expressed in senescent cardiomyocytes
Figure S2 Effects of lncR‐SMAL on cardiac systolic function and senescence
Figure S3 Homology sequence analysis of lncR‐SMAL between human and mouse
Figure S4 Overexpression of lncR‐SMAL inhibits autophagy and causes senescence
Figure S5 Interaction between lncR‐SMAL and Parkin protein in senescent AC16 cells
Figure S6 LncR‐SMAL targets Parkin to regulate cell senescence
Figure S7 Knockout of Parkin cancels the regulation of lncR‐SMAL on autophagy and senescence
Figure S8 BAFA1 blocks the beneficial effects of si‐SMAL on mitophagy and senescence
Figure S9 Knockdown of lncR‐SMAL improves mitophagy
Table S1. Clinical information of patient blood samples
