# Supplementary material for: LncRNA LOC105378097 inhibits cardiac mitophagy in natural ageing mice
Source: Clin Transl Med. 2022 Jun 27;12(6):e908. doi: 10.1002/ctm2.908 (PMC9235350; doi:10.1002/ctm2.908)
Supplement: Supplementary file 1 — Figure S1 LncR‐SMAL is highly specifically expressed in senescent cardiomyocytes Figure S2 Effects of lncR‐SMAL on cardiac systolic function and senescence Figure S3 Homology sequence analysis of lncR‐SMAL between human and mouse Figure S4 Overexpression of lncR‐SMAL inhibits autophagy and causes senescence Figure S5 Interaction between lncR‐SMAL and Parkin protein in senescent AC16 cells Figure S6 LncR‐SMAL targets Parkin to regulate cell senescence Figure S7 Knockout of Parkin cancels the regulation of lncR‐SMAL on autophagy and senescence Figure S8 BAFA1 blocks the beneficial effects of si‐SMAL on mitophagy and senescence Figure S9 Knockdown of lncR‐SMAL improves mitophagy Table S1. Clinical information of patient blood samples [file CTM2-12-e908-s001.docx]

**LncRNA LOC105378097 inhibits cardiac mitophagy in natural aging mice**

**Xin Liu^1,3#^, Xue Bai^1#^, Heng Liu^1#^, Yang Hong^1^, Hao Cui^1^, Lei Wang^1^, Wanqing Xu^1^, Limin Zhao^1^, Xiaohan Li^1^, Huimin Li^1^, Xia Li^1^, Hui Chen^1^, Ziyu Meng^1^, Han Lou^1^, Henghui Xu^1^, Yuan Lin^1^, Zhimin Du^5^, Philipp Kopylov^6^, Baofeng Yang^1.2.3*^, Yong Zhang^1.3.4*^**

^#^With equal contribution to this work.

^1^Department of Pharmacology (the State-Province Key Laboratories of Biomedicine-Pharmaceutics of China, Key Laboratory of Cardiovascular Research, Ministry of Education), College of Pharmacy, Harbin Medical University, Harbin, China.

^2^Department of Pharmacology and Therapeutics, Melbourne School of Biomedical Sciences, Faculty of Medicine, Dentistry and Health Sciences University of Melbourne, Melbourne, Australia.

^3^Research Unit of Noninfectious Chronic Diseases in Frigid Zone, Chinese Academy of Medical Sciences，2019RU070

^4^Institute of Metabolic Disease, Heilongjiang Academy of Medical Science, Harbin, China.

^5^Institute of Clinical Pharmacy, the Second Affiliated Hospital of Harbin Medical University, Harbin, China.

^6^Department of Preventive and Emergency Cardiology, Sechenov First Moscow State Medical University, Moscow, Russian Federation.

**Running title: LncR-SMAL promotes heart aging by targeting Parkin**

**Correspondence:**

Yong Zhang, hmuzhangyong@hotmail.com

Baofeng Yang, yangbf@ems.hrbmu.edu.cn

**Funding**

This study was funded by the National Natural Science Foundation of China (grant number: 91949130, 81961138018, 81903610, 81730012) and HMU Marshal Initiative Funding (HMUMIF-21022).

**Supplementary Figure legends**

**Figure S1 LncR-SMAL is highly specifically expressed in senescent cardiomyocytes**

(A) Expression levels of various lncRNAs in AC16 cells with or without D-gal tested by qRT-PCR; n=5. ***P*<0.01, ****P*<0.001 *vs.* Ctl. (B) Expression level of lncR-SMAL in different organs and tissues of mice treated with or without lncR-SMAL overexpression tested by qRT-PCR; n=5. **P*<0.05, ****P*<0.001 *vs.* Ctl. (C) Expression level of lncR-SMAL in different cell types, including cardiomyocytes (neonatal mouse primary cardiomyocytes), fibroblasts (neonatal mouse primary fibroblasts), endothelial cells (human umbilical vein endothelial cell line, HUVEC) and macrophages (mouse macrophage cell line, RAW264.7); n=5. ****P*<0.001 *vs.* Cardiomyocyte. The data were expressed as the mean ± SD.

**Figure S2 Effects of lncR-SMAL on cardiac systolic function and senescence**

(A) Ejection fraction (EF%) and fractional shortening (FS%) measured by echocardiography; n=5. (B) Senescence-associated secretory phenotype (SASP) TNF-α, IL-6, IL-1, MMP-2 and MMP-9 mRNA levels tested by qRT-PCR; n=5. ***P*<0.01, ****P*<0.001 *vs.* Ctl. The data were expressed as the mean ± SD.

**Figure S3** **Homology sequence analysis of lncR-SMAL between human and mouse**

(A) Detection of partial lncR-SMAL sequence by southern blot in mouse primary cardiomyocytes (219 bp). (B) Identifying amplified sequence from southern blot by ABI3730 Sequencing Instrument and NCBI BLAST database.

**Figure S4 Overexpression of lncR-SMAL inhibits autophagy and causes senescence**

(A) Parkin, p62 and LC3 protein levels in total cell lysate tested by western blot; n=5. ***P*<0.01, ****P*<0.001 *vs.* Ctl. (B) p53 and p21 protein levels tested by western blot; n=5. ****P*<0.001 *vs.* Ctl. The data were expressed as the mean ± SD.

**Figure S5 Interaction between lncR-SMAL and Parkin protein in senescent AC16 cells**

(A) RNA-binding protein immunoprecipitation (RIP) analysis for lncR-SMAL:Parkin interaction in senescent AC16 cells; n=3. ***P*<0.01, compared with groups at both ends of the line. The data were expressed as the mean ± SD.

**Figure** **S6 LncR-SMAL targets Parkin to regulate cell senescence**

(A) EF% and FS% measured by echocardiography; n=5. (B) TNF-α, IL-6, IL-1, MMP-2 and MMP-9 mRNA levels tested by qRT-PCR; n=5. ***P*<0.01, ****P*<0.001, compared with groups at both ends of the line. (C-E) Parkin, p62 and LC3 protein levels in total protein tested by western blot; n=5. **P*<0.05, ***P*<0.01, ****P*<0.001, compared with groups at both ends of the line. (F-G) PINK1 and BNIP3 protein levels in mitochondria tested by western blot; n=5. ****P*<0.001, compared with groups at both ends of the line. The data were expressed as the mean ± SD.

**Figure** **S7 Knockout of Parkin cancels the regulation of lncR-SMAL on autophagy and senescence**

(A-E) Parkin, p62, LC3, p53 and p21 protein levels in total protein tested by western blot; n=3 for p21, n=5 for other proteins. ***P*<0.01, ****P*<0.001 *vs.* D-gal; ^#^*P*<0.05, ^##^*P*<0.01, ^###^*P*<0.001, compared with groups at both ends of the line. (F) Autophagosomes (yellow dots) and autolysosomes (red dots) measured by immunofluorescence staining. Scale bar: 20 μm; n=5. ****P*<0.001 *vs.* D-gal; ^###^*P*<0.001, compared with groups at both ends of the line. (G) Representative images of mito-Keima staining and statistical results of fluorescence intensity (red: 550 nm/green: 440 nm) calculated. Scale bar: 20 μm; n=5. ****P*<0.001 *vs.* D-gal; ^###^*P*<0.001, compared with groups at both ends of the line. The data were expressed as the mean ± SD.

**Figure S8 BAFA1 blocks the beneficial effects of si-SMAL on mitophagy and senescence**

(A-B) p53 and p21 protein levels tested by western blot; n=5. ****P*<0.001 *vs.* D-gal; ^###^*P*<0.001 *vs.* +si-SMAL. (C) Representative images of β-galactosidase staining and statistical results of positive cells. Scale bar: 50 μm; n=5. ****P*<0.001 *vs.* D-gal; ^###^*P*<0.001 *vs.* +si-SMAL. (D-E) LC3 and p62 protein levels tested by western blot; n=5. ****P*<0.001 *vs.* D-gal; ^###^*P*<0.001 *vs.* +si-SMAL. (F) Autophagosomes (yellow dots) and autolysosomes (red dots) measured by immunofluorescence staining. The mean numbers per cell were calculated; Scale bar: 20 μm; n=5. ****P*<0.001 *vs.* D-gal; ^###^*P*<0.001 *vs.* +si-SMAL. The data were expressed as the mean ± SD.

**Figure S9 Knockdown of lncR-SMAL improves mitophagy**

(A) Parkin, p62 and LC3 protein levels in total protein tested by western blot; n=5. **P*<0.05, ***P*<0.01, ****P*<0.001, compared with groups at both ends of the line. (B-C) PINK1 and BNIP3 protein levels in mitochondria tested by western blot; n=5. ***P*<0.01, ****P*<0.001, compared with groups at both ends of the line. (D) Parkin mRNA levels tested by qRT-PCR; n=5. ***P*<0.05, compared with groups at both ends of the line. The data were expressed as the mean ± SD.

**Online Table 1. Clinical information of patient blood samples**

**Supplementary Figures**

**Figure S1**

**
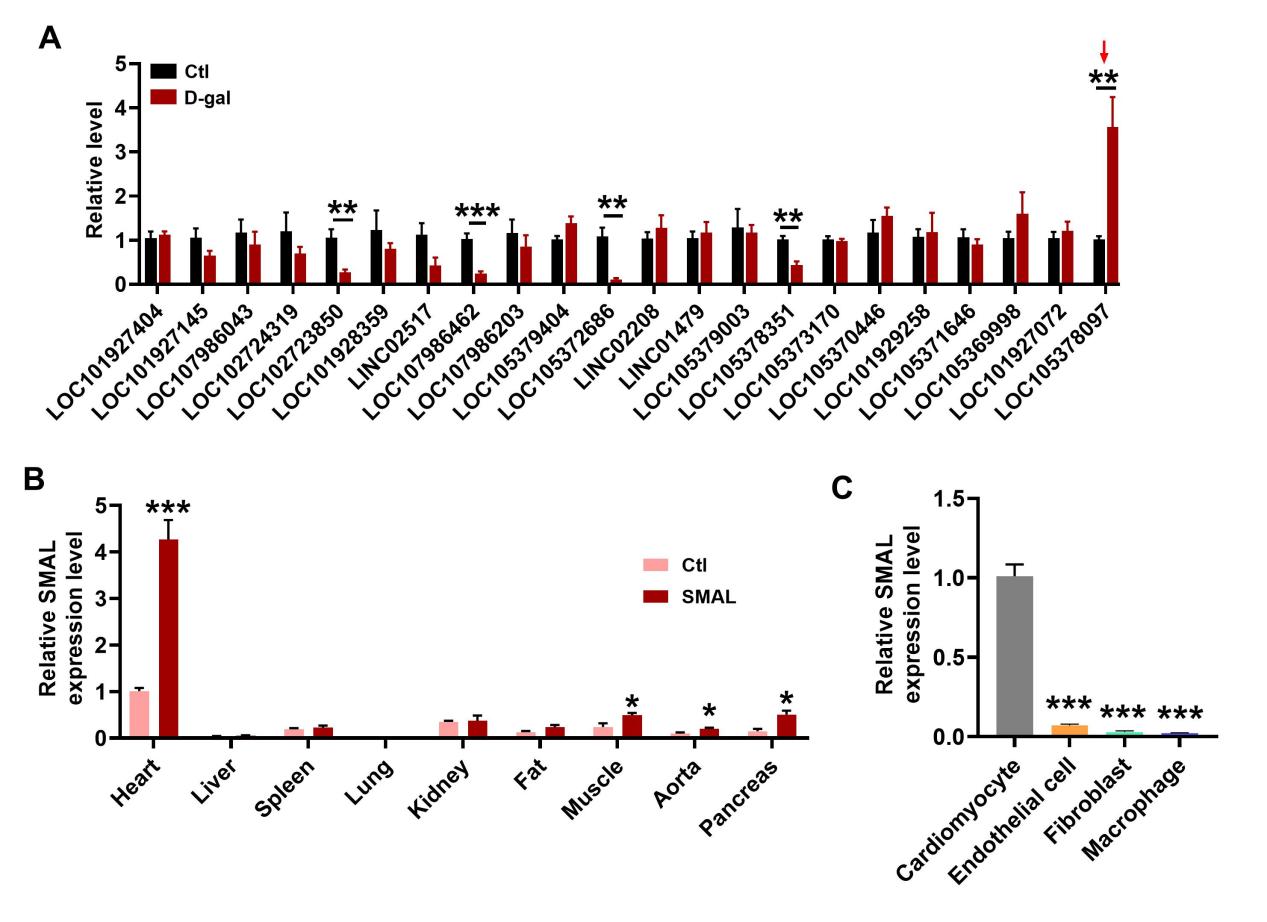
**

**Figure S2**

**
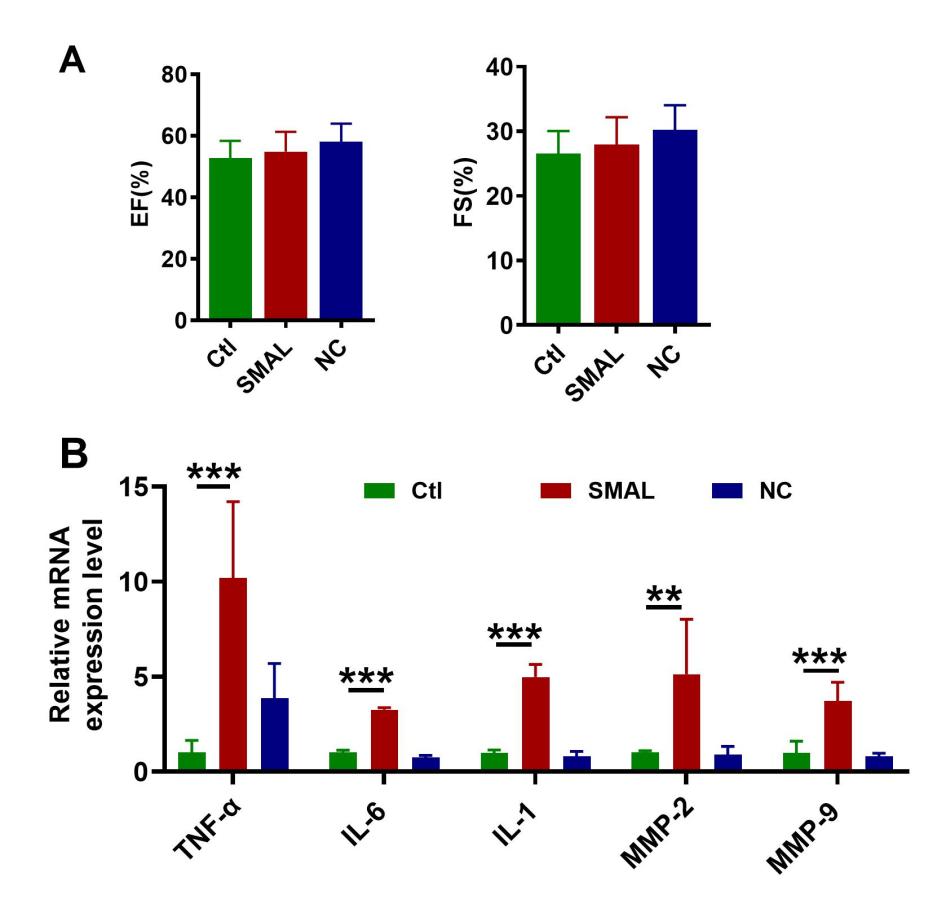
**

**Figure S3**

**
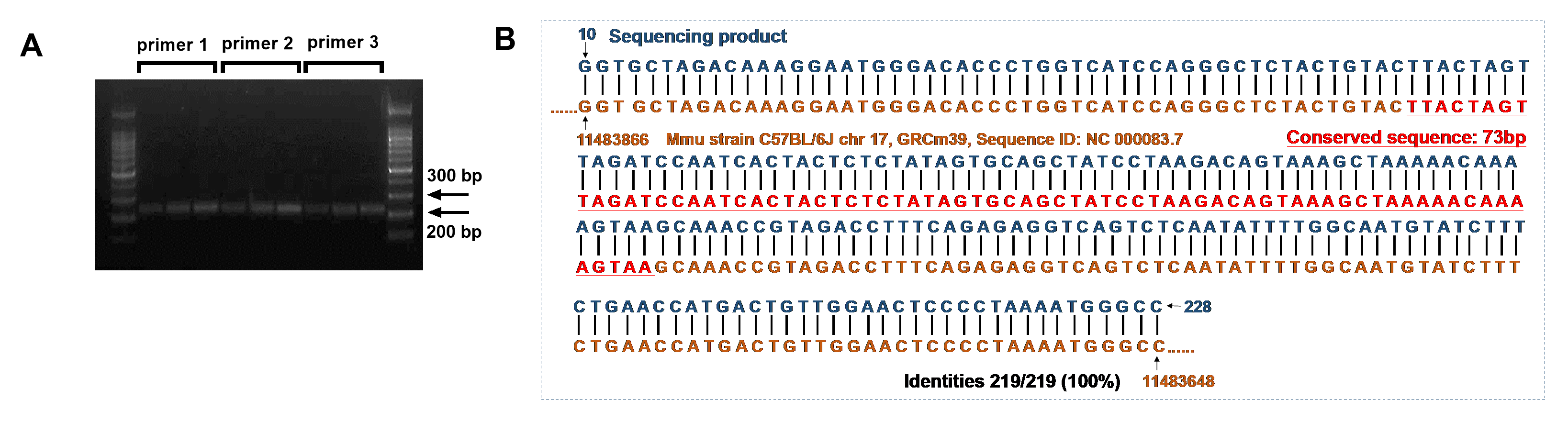
**

**Figure S4**

**
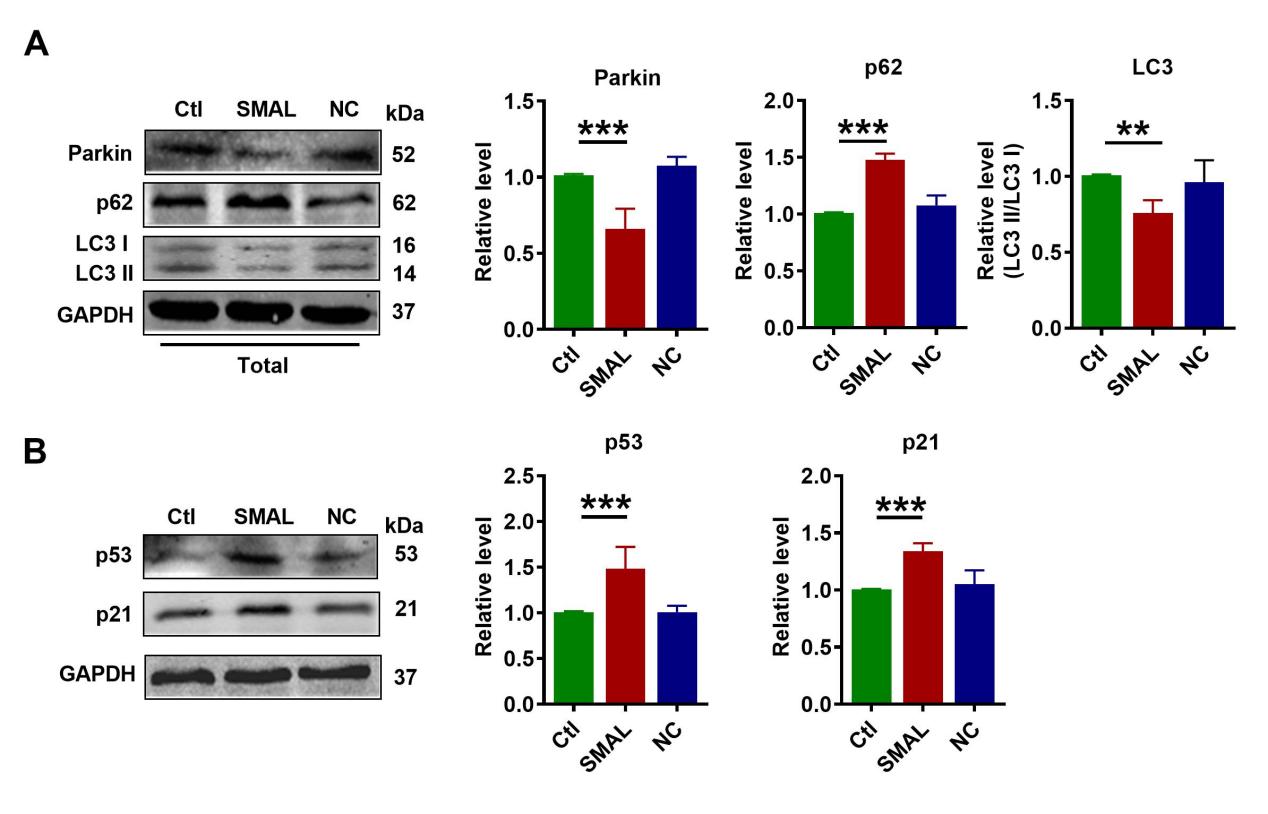
**

**Figure S5**

**
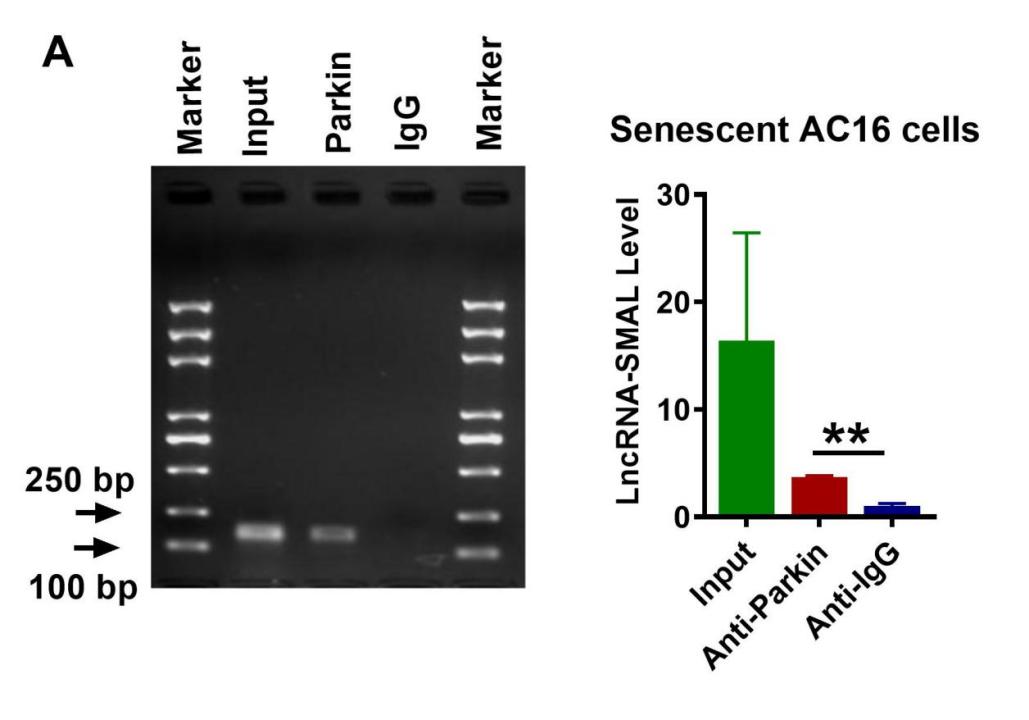
**

**Figure S6**

**
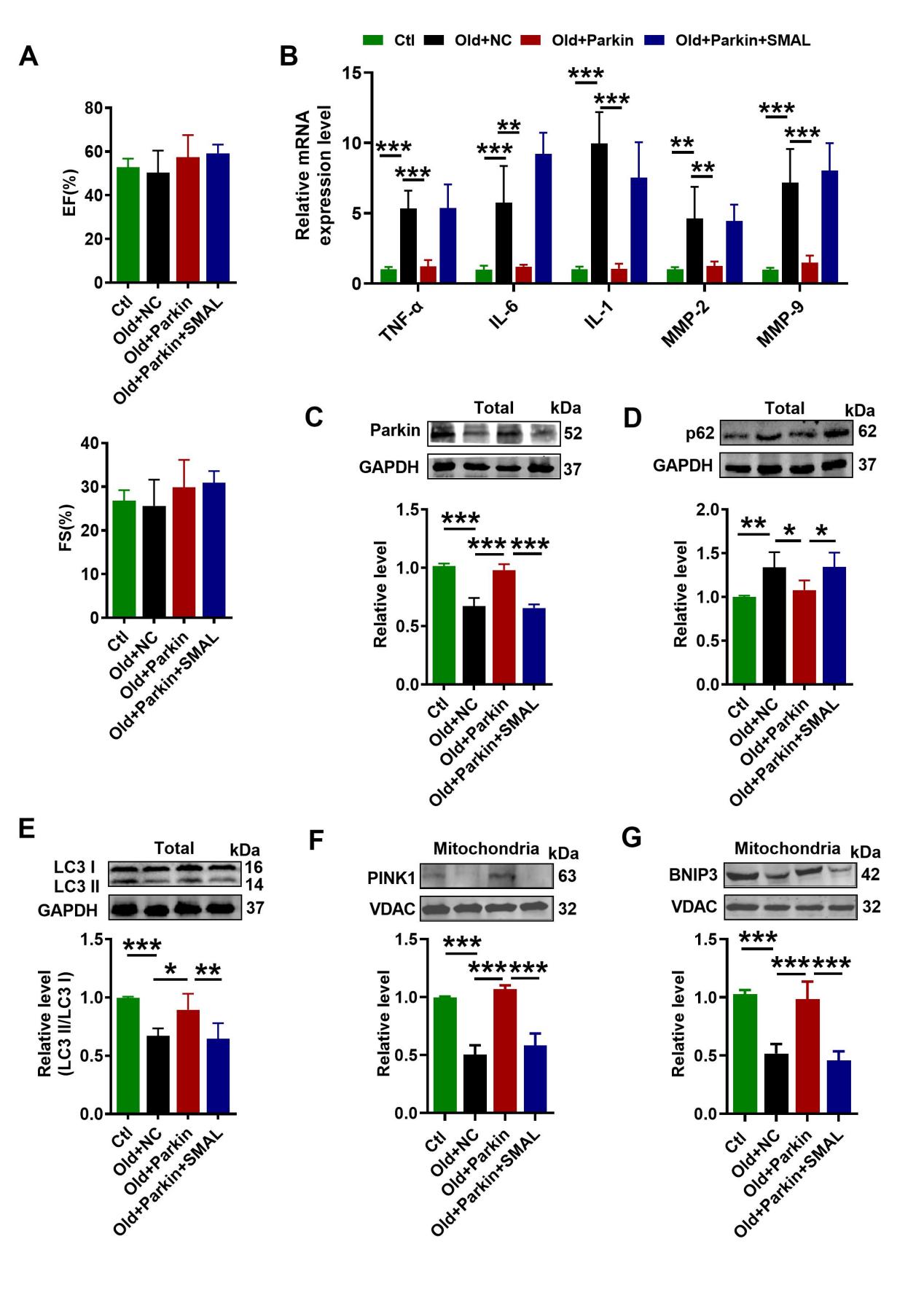
**

**Figure S7**

**
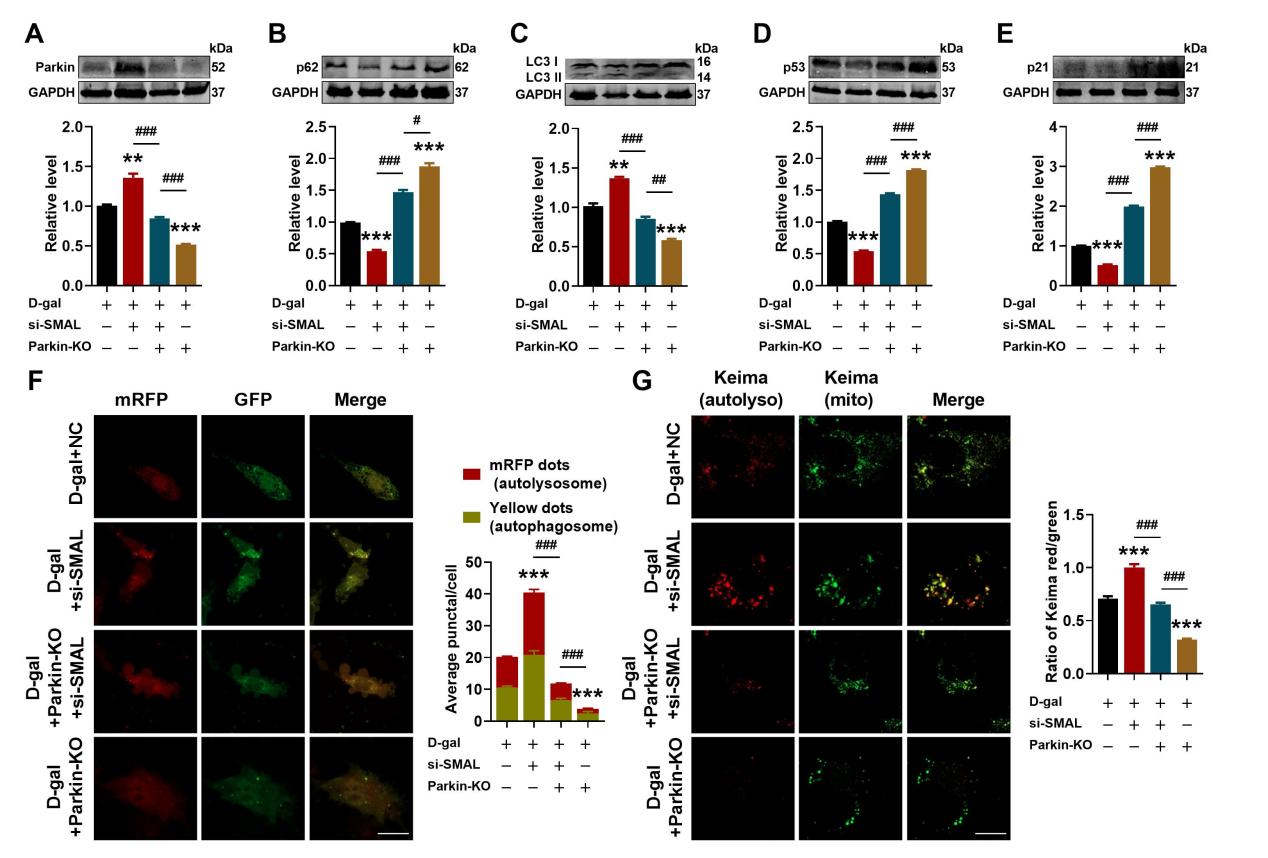
**

**Figure S8**

**
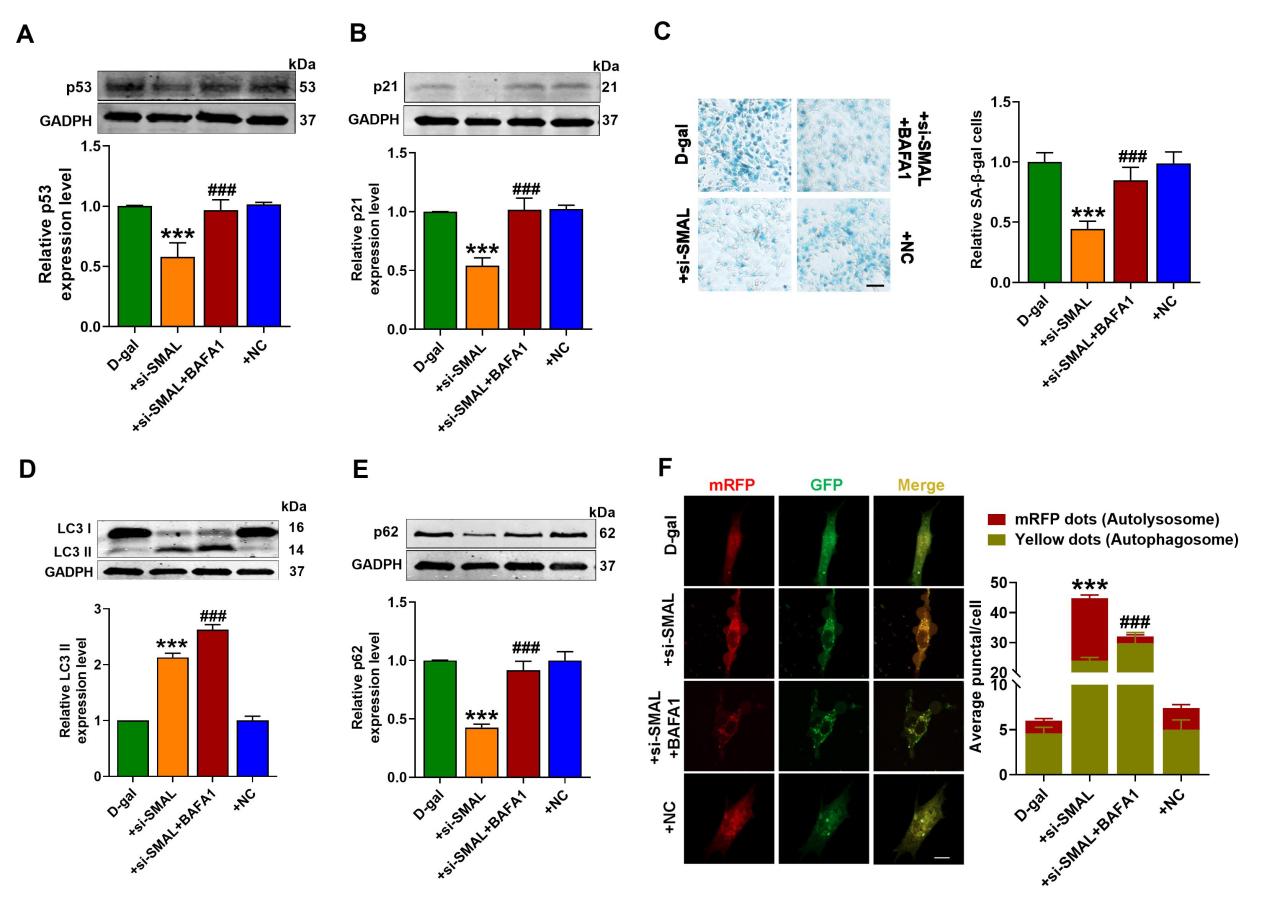
**

**Figure S9**

**
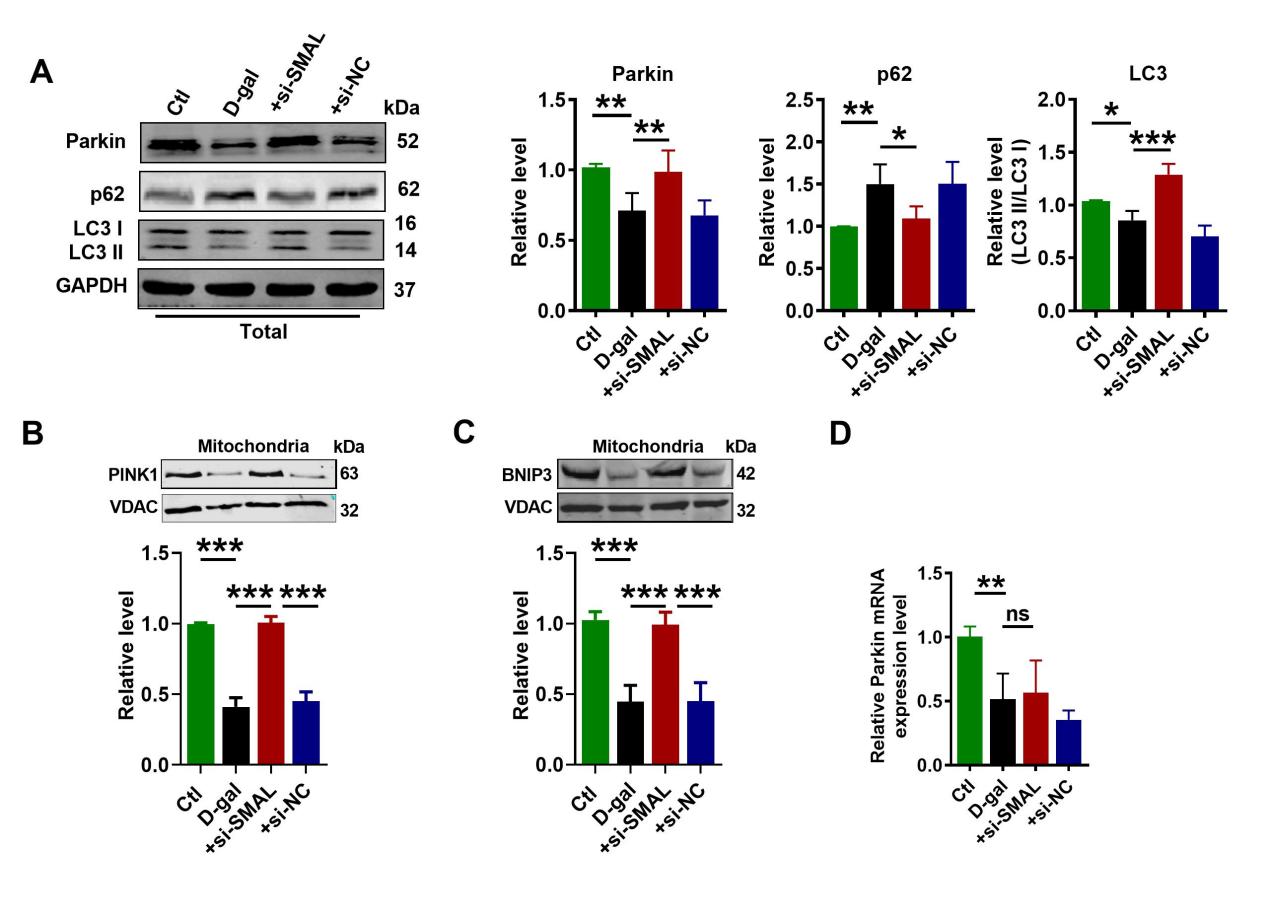
**

**Online Table 1. Clinical information of patient blood samples**

F, female; M, male.

|  | Young  <60  （n=14） | Old  >60  （n=11） |
| --- | --- | --- |
| Age，yrs | 45±11 | 75±7 |
| Gender | M: 8; F: 6 | M: 8; F:3 |
| Body mass index, kg/m^2^ | 21.5±2.3 | 21.1±2.0 |

Values are mean±SD, n (%)
